# Defining Phytochemical Metabolomes of Somatic Hybrids *Gentiana cruciata* L. (+) *G. tibetica* King ex Hook.f. (Gentianaceae) Using UHPLC-DAD-ESI-MS^3^ Analysis in Comparison to the Parental Species

**DOI:** 10.3390/molecules30163321

**Published:** 2025-08-08

**Authors:** Maciej Obrębski, Rafał M. Kiełkiewicz, Karolina Tomiczak, Anita A. Śliwińska

**Affiliations:** 1 MicrobiotaLab, Department of Pharmaceutical Microbiology and Bioanalysis, Faculty of Pharmacy, Medical University of Warsaw, Banacha 1, 02-097 Warsaw, Poland; maciej.obrebski@wum.edu.pl; 2 Department of Pharmaceutical Biology, Faculty of Pharmacy, Medical University of Warsaw, Banacha 1, 02-097 Warsaw, Poland; rafal.kielkiewicz@wum.edu.pl (R.M.K.); anita.sliwinska@wum.edu.pl (A.A.Ś.); 3 Plant Breeding and Acclimatization Institute—National Research Institute in Radzików, Radzików, 05-870 Błonie, Poland

**Keywords:** somatic hybrid plants, cross gentian, Tibetan gentian, in vitro culture, metabolite profiling, secoiridoids, gentiopicroside, biosynthetic potential, metabolomics, LC-MS/MS

## Abstract

Somatic hybridization represents a powerful tool for generating novel chemotypes with enhanced biosynthetic capabilities. This study provides the first comprehensive phytochemical characterization of interspecific somatic hybrids between *Gentiana cruciata* L. and *Gentiana tibetica* King ex Hook.f., two medicinally important yet regionally rare gentians. A total of 107 compounds were detected using UHPLC-DAD-ESI-MS^3^, of which 31 were identified as metabolites across eight phytochemical classes. Comparative profiling revealed that all hybrids retained a conserved core of iridoids and secoiridoids while integrating lineage-specific compounds and producing hybrid-specific metabolites not detected in either parent. Despite inheriting plastids from *G. tibetica*, hierarchical clustering showed that the phytochemical profiles of hybrid lines were more similar to *G. cruciata*, the donor of the nuclear genome. Quantitative analysis of the major secoiridoids, such as gentiopicroside, swertiamarin, and sweroside, demonstrated that several hybrid lines, particularly F30A-5 and F30A-6, matched or surpassed the biosynthetic output of *G. tibetica*, the more productive parent. These lines also exhibited elevated antioxidant capacity, underscoring their phytochemical and functional potential. Altogether, our findings show that somatic hybridization not only preserves but may amplify the secondary metabolite capacity of the parental genotypes, offering a viable platform for sustainable in vitro production of pharmacologically relevant secoiridoids.

## 1. Introduction

*Gentiana* Tourn. ex L. is a cosmopolitan genus from the family Gentianaceae, comprising about 360 species of annual, biennial, or perennial herbs [1]. Numerous species in this genus are distinguished by their ornamental attributes, especially large trumpet-shaped flowers that are often of an intense blue color but can also be white, yellow, or purple [2]. However, much more attention is drawn by the pharmaceutical properties of gentians, as some species have been employed as herbal drugs since ancient times [3,4]. Many of them are still highly popular in traditional medicine, particularly in Asian countries [5,6,7]. Phytochemical investigations of different parts of *Gentiana* species, which started in the 1960s, resulted in the isolation of more than 500 secondary metabolites [4] and demonstrated that this genus is a rich source of iridoids and secoiridoids, xanthones, flavonoids, and triterpenoids [3,6,8,9]. Among iridoids, the loganic acid has been identified as the most remarkable compound, whereas gentiopicroside, amarogentin, amaroswerin, swertiamarine, and sweroside have been the principal secoiridoids [10,11]. Mangiferin, decussatin, gentiacaulein, and isogentisin belong to the most often reported xanthones, while the derivatives of isoorientin and isovitexin are the commonly recognized flavon *C*-glycosides [4]. All the above-mentioned plant-derived molecules possess a broad spectrum of bioactivity, being responsible for antioxidant, anti-inflammatory, anticarcinogenic, antimicrobial, and antidiabetic properties of gentians. Furthermore, they demonstrate cardioprotective, neuroprotective, hepatoprotective, and radioprotective activity [6,9]. Investigations into the isolation and biological evaluation of specialized metabolites in *Gentiana* species remain relevant, particularly in the context of alternative in vitro production systems. For instance, recently, Czarnomska et al. [12] successfully identified novel bioactive compounds from *G. capitata* Buch.-Ham. ex D.Don suspension cultures and demonstrated their cytoprotective potential.

Since numerous gentian species are endemic or endangered due to overexploitation and the destruction of their habitats [9], some of them have been introduced to in vitro culture with the objective of obtaining an efficient source of secondary metabolites. The in vitro culture of plant cells and tissues under controlled conditions can be a feasible strategy for the production of structurally complex and high-value natural products without further depletion of natural populations of plant species or disturbance to their habitats [13,14]. As a result, a number of protocols for micropropagation of different gentian species [15,16,17,18,19], as well as a few for the production of hairy root cultures [20,21,22,23], have been developed. Another approach aimed at the establishment of a novel source of bioactive compounds was somatic hybridization via electrofusion of protoplasts [24,25].

Somatic hybridization is a protoplast-based technology that enables the creation of distinct interspecific and intergeneric hybrids. It can be used as an alternative to traditional sexual hybridization, which is typically limited to closely related species. By combining the nuclear, mitochondrial, and plastid genomes from two different donor plants into one hybrid, following fusion of their protoplasts, it allows for substantial phenotypic variation resulting from unique genetic interactions [26,27]. As a consequence, this process facilitates the creation of novel genotypes with desirable characteristics inherited from parental plant species, in particular polygenic traits, such as tolerance to abiotic stress factors and resistance to pathogens and herbicides, as well as traits encoded by cytoplasmic genomes, such as cytoplasmic male sterility (CMS) [28,29,30,31]. Additional noteworthy objectives of somatic hybridization are the modification of the phenotype of ornamental plants [32,33], the production of seedless fruits [34,35], the enhancement of the production of biologically active compounds [36], and the introgression of the entire biosynthesis pathways into distant hybrids [37,38,39].

The potential of protoplast fusion in the context of medicinal species is highly promising, as somatic hybrids are allopolyploids that combine the characteristics of two distinct species. In recent years, polyploidization has become a powerful tool for the improvement of medicinal and aromatic plants [40,41,42]. However, frequent genetic instability of obtained hybrids, resulting from a genomic shock [43,44], can be a serious limitation. Therefore, it is necessary not only to analyze the hybrid plants phytochemically but also to assess their genetic constitution and to monitor their stability. Since accumulation of active metabolites is both genotype and organ specific, an appropriate selection of suitable plant material is needed as well [45,46].

Recently, we have succeeded in the production of somatic hybrids between *G. kurroo* Royle and *G. cruciata* L. (Cross Gentian) as well as between *G. cruciata* and *G. tibetica* King ex Hook.f. (Tibetan Gentian) [47]. While *G. kurroo* (+) *G. cruciata* plants demonstrated a high degree of genetic instability, as evidenced by a stepwise reduction of total DNA content, poor rooting, and low viability in vitro [25], somatic hybrids between *G. cruciata* and *G. tibetica* were found to be genetically stable and exhibited abundant growth [24]. Both parental species of these hybrids are recognized as medicinal herbs in regions of their occurrence and serve as significant sources of secoiridoids [5,48,49]. Neither species is common; furthermore, *G. cruciata* hosts the larvae of the endangered parasitic butterfly *Phengaris rebeli* [50]. Thus, the necessity to develop an alternative source of secondary metabolites made both species the subject of biotechnological research [16,22,51,52,53,54,55,56]. These efforts, in turn, require equally advanced analytical strategies to investigate the full spectrum of secondary metabolites in both wild and biotechnologically modified plants.

Recent advances in the identification of plant secondary metabolites have been driven by the continuous development of mass spectrometry-based techniques. Modern liquid chromatography–mass spectrometry (LC–MS) techniques now enable the detection of thousands of metabolite signals in a single experiment, owing to ultra-high sensitivity and high-resolution analyzers that can profile both known compounds and previously undetected derivatives [57]. Nevertheless, classical untargeted LC–MS surveys often struggle to confidently identify low-abundance or novel metabolites, while targeted MS assays are inherently restricted to predefined analytes [58]. To overcome these limitations, hybrid approaches now blend the breadth of untargeted profiling with targeted sensitivity, exemplified by widely targeted metabolomics methods. In particular, tandem MS (MS/MS) data allow for the identification of metabolite derivatives based on specific fragmentation pathways and the presence of diagnostic fragment ions, which can reveal structural modifications, such as glycosylation, acylation, or hydroxylation [59]. LC–MS remains a powerful and continually evolving tool in plant metabolite research; for instance, a recent development by Yang et al. (2024) introduced the widely targeted metabolite modificomics strategy, which enables the detection of chemically modified metabolite variants [60].

**Figure 1:**
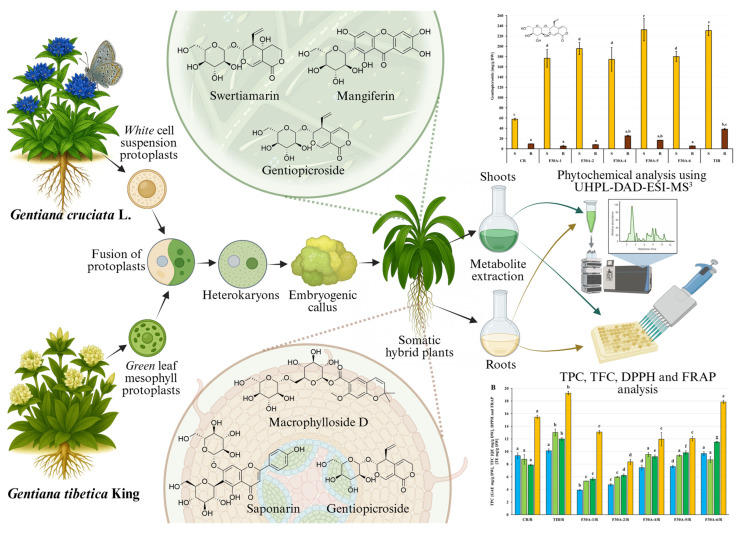
Schematic representation of the investigation conducted within the presented study [61].

The objective of this study was to evaluate the previously obtained somatic hybrids *G. cruciata* (+) *G. tibetica*, with the main goal of comprehensively characterizing the secondary metabolite pool present in shoots and roots in hybrids in comparison to their parents. Moreover, the total phenolic content (TPC), total flavonoid content (TFC), and antioxidant potential of hybrids and their parental species were determined. The study was concluded by evaluating the biosynthetic efficiency of somatic hybrids relative to the parental species, with particular emphasis on the accumulation of the key secoiridoids: gentiopicroside, sweroside, and swertiamarin. The scheme outlining all investigations is summarized in Figure 1.

## 2. Results and Discussion

### 2.1. Comparative Analysis of Secondary Metabolites in Hybrids and Parental Plants

**Figure 2:**
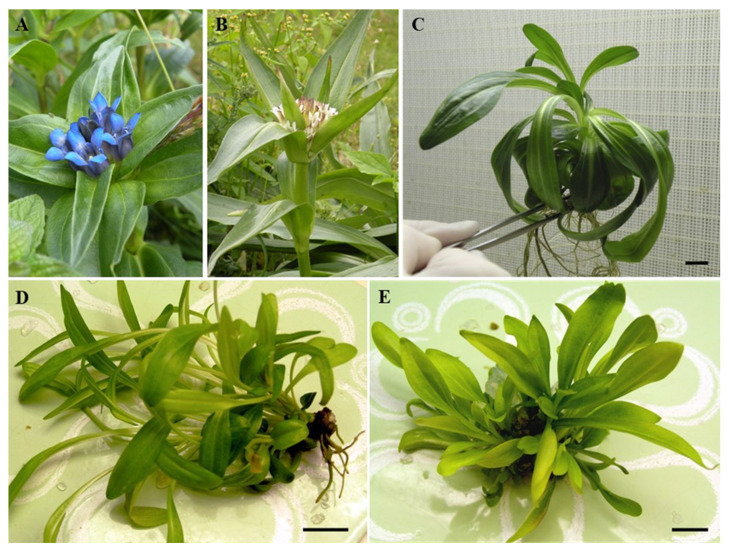
Plant material used in the study: (**A**) parental species *G. cruciata*; (**B**) *G. tibetica* in the collection of the PAS Botanical Garden–CBDC in Powsin, Poland; (**C**) one of the somatic hybrid plants before subculturing; (**D**) shoots of *G. cruciata;* and (**E**) *G. tibetica* from in vitro culture.

**Figure 3:**
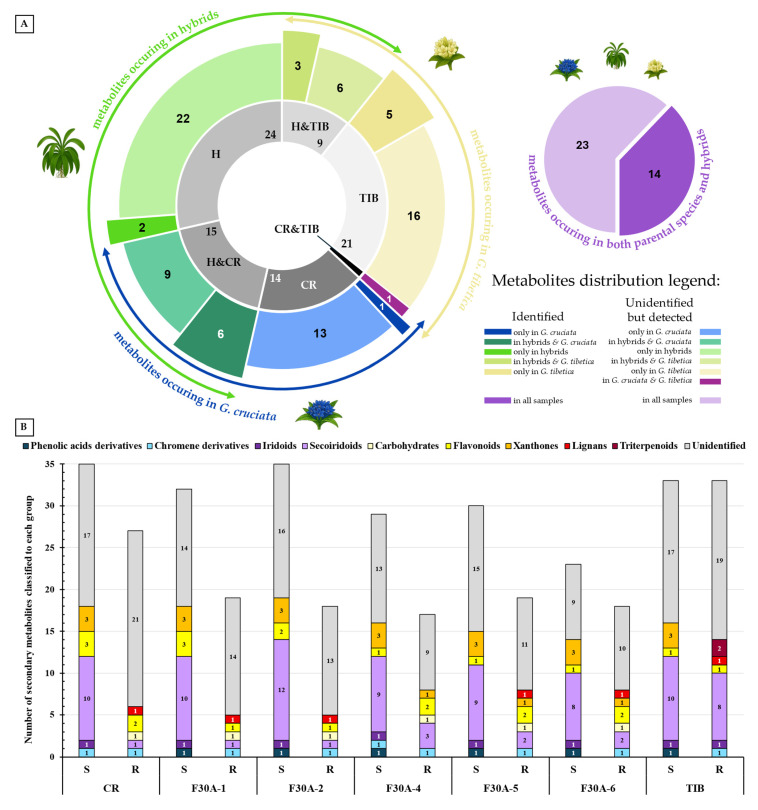
Distribution of secondary metabolites detected using UHPLC-DAD-ESI-MS^3^ analysis in methanolic extracts of *G. cruciata* (CR), *G. tibetica* (TIB), and their somatic hybrid lines (F30A-1 to F30A-6; collectively H). (**A**) Sunburst chart showing the similarity and diversification of secondary metabolites detected, including shared and unique compounds. The inner ring, with the number of total metabolites marked, shows the sample types (CR, TIB, and H) and their intersections—compounds shared by two groups, while the outer ring exhibits the number of identified and unidentified compounds in each group, which correlate with appropriate size and colors described precisely in the legend beside. Additionally, external arrows show parts of the chart representing the total range of metabolites characteristic of particular parent species and for hybrids. The adjacent pie chart indicates the number of compounds detected simultaneously in all three groups (CR ∩ TIB ∩ H), with a breakdown into identified and unidentified metabolites. (**B**) Composition of major chemical classes among identified metabolites in the aerial parts (S) and roots (R) of the analyzed lines. Each stacked column represents the total number of compounds detected in a given sample, while the colored segments within each column indicate the number and relative contribution of metabolites assigned to individual chemical groups.

**Figure 4:**
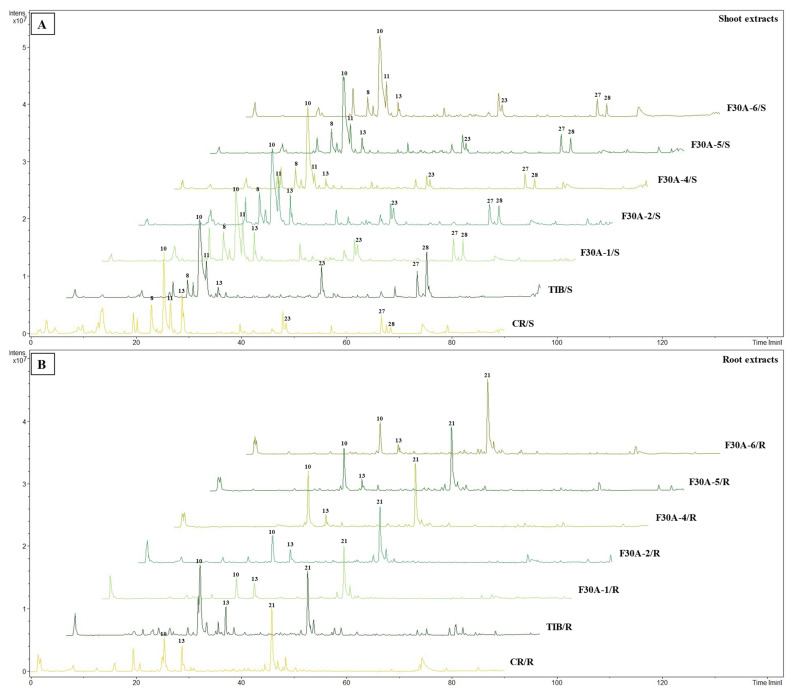
Overlay base peak chromatograms (BPCs) recorded in negative ion mode of shoot (**A**) and root (**B**) extracts from parental species (*G. cruciata* CR and *G. tibetica* TIB) and their somatic hybrids (F30A-1 to F30A-6). The numbers above the peaks on the chromatograms correspond to those listed in Table 1.

**Figure 5:**
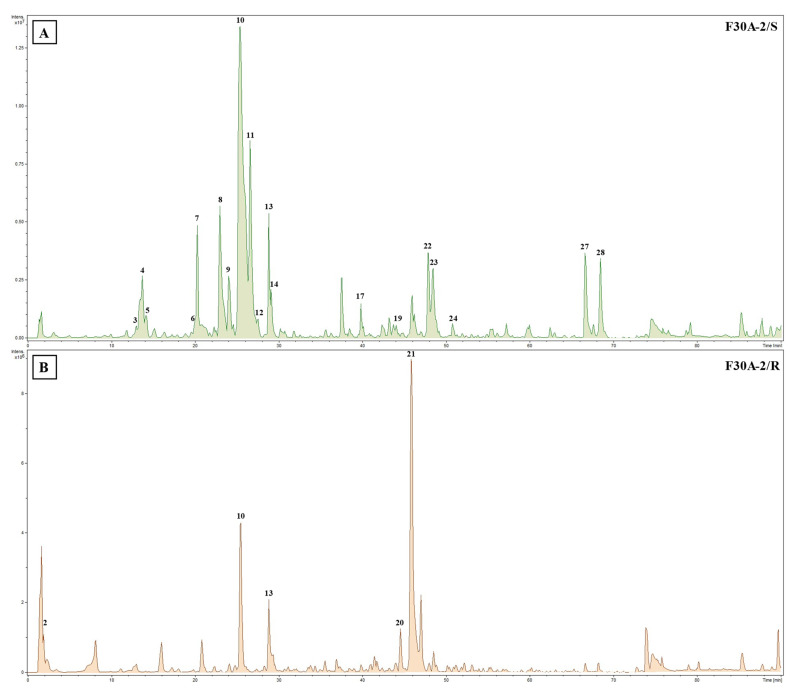
Base peak chromatograms (BPCs) recorded in negative ion mode of shoot (**A**) and root (**B**) extracts from somatic hybrid *G. cruciata* (+) *G. tibetica* F30A-2. The numbers above the peaks on the chromatograms correspond to those listed in Table 1.

**Figure 6:**
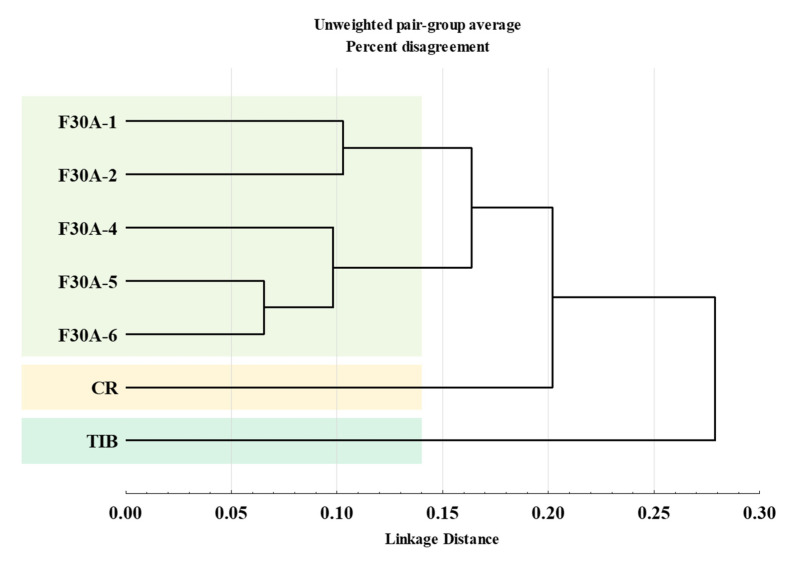
Dendrogram from hierarchical clustering analysis based on binary metabolite presence/absence profiles obtained using data from UHPLC-UV-ESI-MS^3^ qualitative phytochemical analysis determined in extracts of parental plants: *G. cruciata* (CR, “suspension” fusion partner), *G. tibetica* (TIB, “mesophyll” fusion partner), and somatic hybrids (F30A-1, F30A-2, F30A-4, F30A-5, and F30A-6). Clustering was performed using the unweighted pair–group method with arithmetic mean (UPGMA) and percent disagreement as the distance metric.

**Figure 7:**
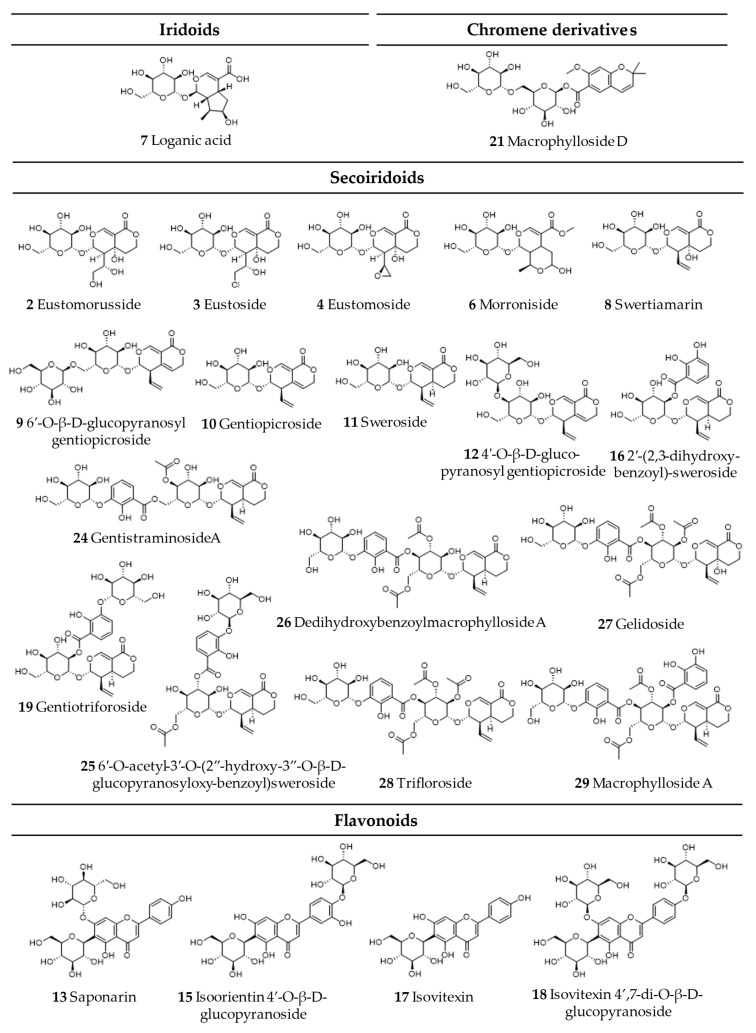

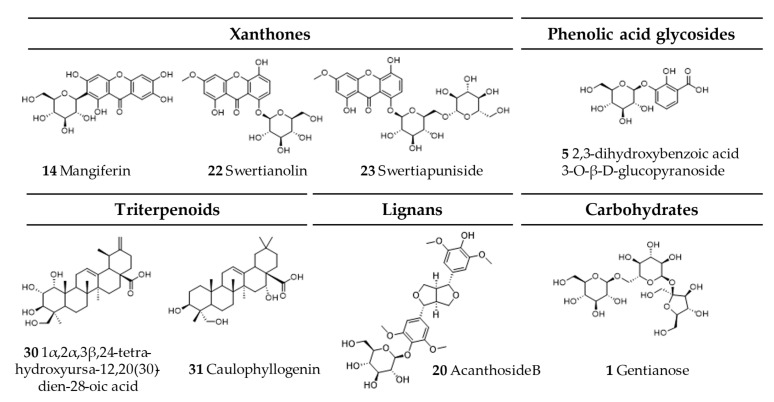
Chemical structures of all identified secondary metabolites in *G. cruciata* (+) *G. tibetica* hybrids and parental species.

Comprehensive analysis of methanolic extracts obtained from shoots (S) or roots (R) of in vitro cultivated parental plants—*G. cruciata* (CR, “suspension” fusion partner, Figure 2A,D)*, G. tibetica* (TIB, “leaf mesophyll” fusion partner, Figure 2B,E), and five somatic hybrids (F30A-1, F30A-2, F30A-4, F30A-5 and F30A-6, Figure 2C)—performed using UHPLC-DAD-ESI-MS^3^ revealed the presence of a total of 107 secondary metabolites. Among them, 31 were identified and classified into nine groups: iridoids, secoiridoids, flavonoids, xanthones, lignans, triterpenes, chromene derivatives, phenolic acid glycosides, and carbohydrates. The distribution of the detected metabolites into particular groups against all the identified compounds in each extract is presented in Figure 3. Base peak chromatograms (BPCs) recorded in negative ion mode for all extracts are overlayed in Figure 4. Additionally, chromatograms of one representative of the somatic hybrids, F30A-2, illustrating the phytochemical profile of its shoots and roots, are presented in Figure 5. Peak numbers corresponding to the tentatively identified compounds are presented in Table 1 in order of the elution time. Due to the large number of detected compounds, a table containing all detected compounds and their fragmentation patterns is provided in the supplement, in Table S1. Moreover, in the column marked “C/T”, it was indicated whether a given compound has been previously reported in *G. cruciata* (C) and/or *G. tibetica* (T) according to literature sources. Notably, the dendrogram generated using UPGMA clustering and percent disagreement as the distance metric (Figure 6) was constructed based on the complete binary matrix of compound presence/absence derived from Table S1, which includes both identified and unidentified secondary metabolites. The chemical structures of all identified compounds, grouped by metabolite classes, are presented in Figure 7.

Regarding the results of the qualitative UHPLC-DAD-ESI-MS analysis shown in Table 1, the number and diversity of secondary metabolites differed notably between shoots and roots as well as among parental species and somatic hybrid lines. However, some crucial tendencies can be highlighted (Figure 3B). In both parental species, CR and TIB, shoots accumulated a greater number of compounds compared to roots. This pattern was consistently observed in all somatic hybrids as well, which reflects tissue-specific expression of biosynthetic pathways, with aerial parts being more active in the production of various secondary metabolites than roots. In all analyzed samples, the predominant class of secondary metabolites was secoiridoids, with the highest representation in the shoots of hybrid F30A-2 (12 identified compounds), which is the only one ahead of both parental species (each with 10 compounds identified). Furthermore, a trend can be observed in both hybrids and parents, in which the shoots of the analyzed plants were found to be much richer and more diverse in terms of secoiridoids than the roots. Similarly, the identified xanthones were also present in all shoots in comparison to roots (only three root samples). The number of flavonoids identified in shoots vs. roots varied between species and hybrids, while the only identified representative of phenolic acid derivatives was observed exclusively in shoots of TIB and hybrids. While iridoids were consistently detected across all analyzed extracts, lignans and triterpenoids exhibited strict organ specificity, being present only in root samples.

The aforementioned somatic hybrid F30A-2 was the only line that exhibited a higher overall number of detected metabolites in shoots compared to both parental species. This may suggest additive or complementary biosynthetic capacities arising, which rarely occur, mainly due to the random character of the hybridization process, and the substantial loss of genetic material observed in our previous study, being a result of natural recombination triggered by genomic shock following protoplast fusion [24]. At first glance, the comparison of identified metabolites might suggest that the phytochemical biosynthetic potential of somatic hybrids is substantially reduced compared to the parental species. This impression stems from the lower number of annotated compounds in most hybrid lines relative to CR and TIB. However, this view is incomplete and potentially misleading when not considering the full metabolite dataset, including unidentified secondary metabolites.

When the analysis is extended to encompass both identified and unknown compounds, based on pseudomolecular ion patterns, retention times, and UV spectra presented in Table S1, it becomes evident that a considerable number of unidentified metabolites are present exclusively in somatic hybrids and absent in the parental lines. As illustrated in Figure 3A, among all compounds detected in the dataset, 14 and 21 were unique to *G. cruciata* and *G. tibetica*, respectively, while 24 were found exclusively in hybrid lines. Among them, two metabolites—morroniside and gentiotrifloroside—were identified in the shoots of several hybrid lines (in particular, F30A-1 and F30A-2 in both compounds; and F30A-5 in gentiotrifloroside only) and were entirely absent from *G. cruciata* and *G. tibetica*. Interestingly, both compounds have been previously reported in other *Gentiana* species (*G. septemfida* Pall. [7], *G. straminea* Maxim. [62], *G. triflora* Pall. and *G. rigescens* Franch. [63]), but not in either of the parental taxa studied here, and were not observed in their extracts at detectable levels. This observation suggests that their presence in somatic hybrids may reflect the activation of cryptic or low-expression biosynthetic pathways inherited from either parent, now transcriptionally reprogrammed in the hybrid context. These compounds are therefore proposed as part of a putative hybrid-specific metabolic signature. Although the structure of unidentified hybrid-specific compounds remains unresolved, their presence across multiple hybrid lines, or conversely in a single sample only, provides evidence of novel or reprogrammed biosynthetic activity induced by somatic fusion. This highlights the importance of non-annotated metabolites in assessing metabolic diversity.

Conversely, however, a notable subset of compounds detected in the parental species was entirely absent from all somatic hybrid lines, suggesting partial loss or silencing of specific biosynthetic pathways. Specifically, six metabolites [i.e., eustomorusside was present only in CR shoots, and the compounds present in shoots or roots of TIB included 6′*-O-*acetyl-3′*-O-*(2”-hydroxy-3”*-O-*β-D-glucopyranosyloxybenzoyl)-sweroside, dedihydroxybenzoate-macrophylloside A, macrophylloside A, 1α, 2α, 3β, 24-tetrahydroxyursa-12, 20(30)-dien-28-oic acid, and caulophyllogenin] were not detected in any of the somatic hybrid lines, despite their clear presence in one of the parental phytochemical profiles. Their absence across all six hybrids suggests potential silencing or epigenetic suppression of specific biosynthetic pathways, genomic loss, or a lack of compatibility in regulatory networks following somatic fusion during hybrid formation. The selective absence of these compounds underscores the non-additive and complex nature of metabolomic inheritance in somatic hybrids and further supports the hypothesis of genomic restructuring leading to incomplete parental trait retention. Further targeted analyses are warranted to determine whether these compounds fall below detection thresholds or are indeed absent due to genomic or regulatory suppression.

Furthermore, the majority of other metabolites exhibited inheritance patterns indicative of parental origin, thereby supporting a mosaic-like distribution of metabolic traits in somatic hybrids. Concurrently, 14 compounds identified were shared across all samples [loganic acid, swertiamarin, 6′*-O-*β-D-glucopyranosyl gentiopicroside, gentiopicroside (syn. gentiopicrin), sweroside, 4′*-O-*β-D-glucopyranosyl gentiopicroside, saponarin (syn. isovitexin-7*-O-*β-D-glucopyranoside), mangiferin, acanthoside B (syn. eleutheroside E1; syringaresinol 4*-O-*β-D-glucopyranoside), macrophylloside D, swertianolin, swetiapuniside, gelidoside (syn. rindoside), trifloroside], while structures of an additional 23 could not be elucidated.

Suggested enhanced metabolomic diversity resulting from somatic hybridization may be explained by metabolic complementation between the parental genomes, which share overlapping but non-identical biosynthetic pathways. This potentially leads to the activation of previously silent genes and the formation of entirely new metabolite profiles [64]. Our analysis confirmed that *G. cruciata* and *G. tibetica* possess similar, yet distinct, specialized metabolite profiles. Following somatic fusion, the resulting hybrids not only acquire an expanded gene pool (2n = 88 or 2n = 90 vs. 2n = 52 in both parents) but also undergo genomic restructuring, including cross-hybridization between parental chromosomes [24]. These complex nuclear and cytoplasmic interactions may drive regulatory reprogramming and de novo biosynthetic activity. Altogether, genomic and biochemical complexity provides a strong rationale to postulate that novel, previously unreported secondary metabolites may arise as a direct outcome of somatic hybridization, highlighting the importance of their thorough phytochemical characterization and biological evaluation. Orians et al. [65] reported that novel metabolites, compounds produced exclusively by hybrid plants and absent in both parents, are often present at a frequency of *ca* 27% of the hybrid metabolome across diverse plant taxa, while Cheng reported only 5.5% [66]. In our study, approximately 22.5% of all detected compounds were found exclusively in the somatic hybrids and not in either parental species.

Thus, the identified metabolite pool provides only a partial picture of the phytochemical reality (Figure 3B); inclusion of the unknown yet consistently detectable secondary metabolites is essential to fully appreciate the metabolic complexity of the hybrids. These data were also used to construct a phytochemical similarity matrix, which served as the basis for hierarchical clustering (UPGMA). The resulting dendrogram, illustrating the relationships among all analyzed samples based on their metabolite profiles, is presented in Figure 6.

The dendrogram generated using UPGMA clustering and percent disagreement as the distance metric (Figure 6) reveals distinct groupings among the parental species and somatic hybrids based on the qualitative presence/absence of detected secondary metabolites. The somatic hybrids (F30A-1, F30A-2, F30A-4, F30A-5 and F30A-6) clustered together into a compact group, well separated from both parental genotypes. This pattern shows that the hybrid lines share a common and relatively homogeneous phytochemical signature that is distinct from either parent. Within the hybrid group, F30A-5 and F30A-6 appeared most similar, forming a tight subcluster. The close phytochemical similarity observed between F30A-5 and F30A-6 may be explained by their shared developmental timing and origin, as both lines were regenerated from the same hybrid callus, approximately six weeks later than the other somatic hybrids. In our previous study, we demonstrated that the grouping of somatic hybrids by genetic alterations reflected the influence of regeneration timing and culture age on genomic stability, in line with literature reporting the accumulation of genetic alterations in prolonged in vitro cultures [24]. The current metabolomic analysis reinforces these observations, as the hybrid lines cluster in a similar pattern, suggesting that metabolic profiles may also be shaped by the similar underlying genomic dynamics.

The parental plants CR and TIB remained isolated, each forming its own separate branches. The larger linkage distance observed between the hybrids and *G. tibetica* indicates a more divergent metabolite profile, despite TIB being the source of the mesophyll protoplast. Notably, the somatic hybrids exhibited a phytochemical pattern more closely aligned with CR, the donor of the cell suspension-derived protoplasts. The average linkage distance between the hybrids and TIB was approximately 0.27, while the distance to CR was notably shorter, at ~0.19. A similar trend was observed in our earlier molecular characterization, where AFLP and ISSR molecular marker analysis revealed that hybrid lines retained a greater proportion of *G. cruciata*-specific bands, while a larger fraction of *G. tibetica*-derived markers were eliminated [24]. Importantly, all hybrid lines displayed a CR-dominant nuclear DNA profile while retaining TIB-type plastid genomes. This organelle-nuclear combination may underlie the observed convergence in metabolite patterns among the hybrid lines, potentially favoring plastidial biosynthetic pathways, such as the MEP-dependent secoiridoid route [67].

Nevertheless, both dendrograms, based on molecular markers [24] and phytochemical profiles (present study), revealed consistent clustering patterns. This consistency between genotypic and metabolomic similarity supports the hypothesis of a dominant influence of the CR nuclear genome on hybrid phenotype, reflecting the assumed dominant influence of the “suspension” fusion partner nuclear genome on secondary metabolism.

Subsequent paragraphs provide a detailed overview of the metabolite groups detected, focusing on their relative abundance and distribution across the parental lines and somatic hybrids.

**Table 1:** Qualitative phytochemical profiling of methanolic extracts from shoots (S) and roots (R) of parental species (*G. cruciata—*CR and *G. tibetica—*TIB) and their somatic hybrids (F30A-1 to F30A-6), obtained using UHPLC-DAD-ESI-MS^3^ analysis. The table lists retention times (t_R_), UV-Vis absorption maxima, deprotonated pseudomolecular ions ([M − H]^−^), major MS^2^ and MS^3^ fragment ions, as well as compound occurrence (+) across the analyzed samples. The column marked “C/T” indicates whether a given compound has been previously reported in *G. cruciata* (C) and/or *G. tibetica* (T) according to literature sources.

No.	Compound	Metabolite Group	t_R_ [min]	UV-Vis [nm]	[M−H]¯ *m*/*z*	MS^2^ Ions *m*/*z*	MS^3^ Ions *m*/*z*	CR		F30A-1		F30A-2		F30A-4		F30A-5		F30A-6		TIB		Previously Reported in C/T	Ref.
								**S**	**R**	**S**	**R**	**S**	**R**	**S**	**R**	**S**	**R**	**S**	**R**	**S**	**R**		
**1**	Gentianose	Carbohydrate	2.0	-	503	549 [M − H + HCOOH]^−^ 341 [M − H − Frc]^−^ 179 [M − H − Frc − Glc]^−^			**+**		**+**		**+**		**+**		**+**		**+**				[68]
**2**	Eustomorusside	Secoiridoid	3.1	-	407	453 [M − H + HCOOH]^−^ 329 245 [M − H − Glc]^−^ 209 [M − H − Glc − H_2_O − H_2_O]^−^		**+**															[69]
**3**	Eustoside	Secoiridoid	13.0	-	425	471 [M − H + HCOOH]^−^ 263 [M − H − Glc]^−^ 208, 141		**+**				**+**											[6]
**4**	Eustomoside	Secoiridoid	13.8	-	389	435 [M − H + HCOOH]^−^ 227 [M − H − Glc]^−^ 209 [M − H − Glc − H_2_O]^−^ 141		**+**		**+**		**+**		**+**		**+**		**+**				C	[6,70]
**5**	2,3-dihydroxybenzoic acid 3-*O*-*β*-D-glucopyranoside	Phenolic acid glycoside	14.3	308	315	153 [M − H − Glc]^−^				**+**		**+**		**+**		**+**		**+**		**+**			[71]
**6**	Morroniside	Secoridoid	20.1	-	405	451 [M − H + HCOOH]^−^ 243 [M − H − Glc]^−^				**+**		**+**											[6,72]
**7**	Loganic acid	Iridoid	20.3	-	375	323 213 [M − H − Glc]^−^ 169 [M − H − Glc − CO_2_]^−^		**+**		**+**		**+**		**+**		**+**		**+**		**+**	**+**	C,T	[6,73]
**8**	Swertiamarin	Secoiridoid	23.0	-	373	419 [M − H + HCOOH]^−^ 211 [M − H − Glc]^−^ 179 [Glc − H]^−^ 161 [Glc − H − H_2_O]^−^		**+**		**+**		**+**		**+**		**+**		**+**		**+**	**+**	C,T	[6,49,73]
**9**	6′-*O*-*β*-D-glucopyranosyl gentiopicroside	Secoiridoid	24.1	-	517	563 [M − H + HCOOH]^−^ 341 [2Glc + H_2_O − H]^−^ 323 [2Glc − H]^−^ 281, 251 193 [M − H − 2Glc]^−^ 179 [Glc − H]^−^		**+**		**+**		**+**		**+**		**+**		**+**		**+**	**+**	T	[72,74]
**10**	Gentiopicroside (syn. gentiopicrin)	Secoiridoid	25.3	274 243	355	401 [M − H + HCOOH]^−^ 225 193 [M − H − Glc]^−^ 179 [Glc − H]^−^ 143 [Glc − H − 2H_2_O]^−^ 119 [Glc − H − (CHOH)_2_]¯		**+**	**+**	**+**	**+**	**+**	**+**	**+**	**+**	**+**	**+**	**+**	**+**	**+**	**+**	C,T	[6,49,73,75]
**11**	Sweroside	Secoiridoid	26.6	244	357	403 [M − H + HCOOH]^−^ 267 [M − H − (CHOH)_3_]^−^ 195 [M − H − Glc]^−^ 179 [Glc − H]^−^ 161 [Glc − H − H_2_O]^−^ 125		**+**		**+**		**+**		**+**		**+**		**+**		**+**	**+**	C,T	[6,49,73]
**12**	4′-*O*-*β*-D-glucopyranosyl gentiopicroside	Secoiridoid	27.5	-	517	563 [M − H + HCOOH]^−^ 355 [M − H − Glc]^−^ 341 [2Glc + H_2_O − H]^−^ 179 [Glc − H]^−^		**+**		**+**		**+**		**+**		**+**		**+**		**+**			[74]
**13**	Saponarin (syn. isovitexin-7-*O*-*β*-D-glucopyranoside)	Flavonoid	28.8	214, 270	593	503 [M − H − (CHOH)_3_]^−^ 473 [M − H − (CHOH)_4_]^−^ 431 [M − H − Glc]^−^ 341 [M − H − Glc − (CHOH)_3_]^−^ 311 [M − H − Glc − (CHOH)_4_]^−^ 283		**+**	**+**	**+**	**+**	**+**	**+**	**+**	**+**	**+**	**+**	**+**	**+**	**+**	**+**	C,T	[6,72]
**14**	Mangiferin	Xanthone	29.1	257, 319	421	403 [M − H − H_2_O]^−^ 331 [M − H − (CHOH)_3_]^−^ 301 [M − H − (CHOH)_4_]^−^ 259 [M − H − Glc]^−^		**+**		**+**		**+**		**+**		**+**		**+**		**+**		C	[6,49,72,73]
**15**	Isoorientin 4′-*O*-*β*-D-glucopyranoside	Flavonoid	29.2	227	609	447 [M − H − Glc]^−^ 357 [M − H − Glc − (CHOH)_3_]^−^ 327 [M − H − Glc − (CHOH)_4_]^−^ 299			**+**						**+**		**+**		**+**				[6,72]
**16**	2′-(2,3-dihydroxybenzoyl)sweroside	Secoiridoid	31.9	-	493	539 [M − H + HCOOH]^−^ 339 [M − H − doBA − H_2_O]^−^ 179 [Glc − H]^−^								**+**	**+**		**+**		**+**		**+**	T	[48]
**17**	Isovitexin	Flavonoid	39.9	230	431	413 [M − H − H_2_O]^−^ 341 [M − H − (CHOH)_3_]^−^ 311 [M − H − (CHOH)_4_]^−^ 283		**+**		**+**		**+**										T	[6]
**18**	Isovitexin 4′,7-di-*O*-*β*-D-glucopyranoside (syn. saponarin 4′-*O*-*β*-D-glucopyranoside)	Flavonoid	42.4	216	755	593 [M − H − Glc]¯ 431 [M − H − 2Glc]^−^	311 [M − H − 2Glc − (CHOH)_4_]¯	**+**		**+**												C	[49]
**19**	Gentiotrifloroside (syn. 2-*O*-(2-hydroxy-3-*O*-*β*-D-glucopyranosyl-oxybenzoyl)sweroside)	Secoiridoid	44.1	220	655	493 [M − H − Glc]^−^ 195 [M − H − Glc − doBA − Glc]^−^				**+**		**+**				**+**							[72]
**20**	Acanthoside B (syn. eleutheroside E1; syringaresinol 4-*O*-*β*-D-glucopyranoside)	Lignan	44.6	231	579	417 [M − H − Glc]^−^ 181, 166			**+**		**+**		**+**				**+**		**+**		**+**		[68]
**21**	Macrophylloside D	Chromene derivative	45.9	242, 324	557	603 [M − H + HCOOH]^−^ 593 [M − H + HCl]^−^ 437, 369 359 [M − H − Glc − 2H_2_O]^−^ 323, 263, 247, 233 [M − H − 2Glc]^−^ 221 179 [Glc − H]^−^		**+**	**+**		**+**		**+**	**+**	**+**		**+**		**+**		**+**	T	[76]
**22**	Swertianolin	Xanthone	47.9	210, 310	435	417 [M − H − H_2_O]^−^ 315 [M − H − (CHOH)_3_]^−^ 287 273 [M − H − Glc]^−^ 197, 187, 137	229 [M − H − Glc − CO_2_]^−^ 185 [M − H − Glc − 2CO_2_]^−^	**+**		**+**		**+**		**+**		**+**		**+**		**+**			[77]
**23**	Swetiapuniside	Xanthone	48.5	233	597	435 [M − H − Glc]^−^	315, 299, 297 273 [M − H − 2Glc]^−^ 229 [M − H − 2Glc − CO_2_]^−^ 195, 153, 137	**+**		**+**		**+**		**+**	**+**	**+**	**+**	**+**	**+**	**+**			[78]
**24**	Gentistraminoside A (syn. 4′-*O*-acetyl-6′-*O*-(2”-hydroxy-3”-*O*-*β*-D-glucopyranosyloxybenzoyl)-sweroside)	Secoiridoid	50.8	219	697	655 [M − H − Ac]^−^ 571 535 [M − H − Glc]^−^ 409	475 [M − H − Glc − Ac − H_2_O]^−^ 357 [M − H − Glc − doBA − Ac]^−^ 349, 153					**+**								**+**			[72,79]
**25**	6′-*O*-acetyl-3′-O-(2”-hydroxy-3”-*O*-*β*-D-glucopyranosyloxybenzoyl)-sweroside	Secoiridoid	51.1	231	697	743 [M − H + HCOOH]^−^ 535 [M − H − Glc]^−^															**+**		[6,72]
**26**	Dedihydroxybenzoate-macrophylloside A	Secoiridoid	59.8	232	739	697 [M − H − Ac]^−^ 577 [M − H − Glc]^−^ 535 [M − H − Glc–Ac]^−^	451													**+**			[72]
**27**	Gelidoside (syn. rindoside)	Secoiridoid	66.6	233	797	755 [M − H − Ac]^−^ 713 [M − H − 2Ac]^−^ 635 [M − H − Glc]^−^	593 [M − H − Glc − Ac]^−^ 373 [M − H − Glc − doBA − 3Ac]^−^	**+**		**+**		**+**		**+**	**+**	**+**		**+**		**+**	**+**	T	[6,72,79,80]
**28**	Trifloroside	Secoiridoid	68.4	237	781	739 [M − H − Ac]^−^ 619 [M − H − Glc]^−^ 577 [M − H − Glc − Ac]^−^ 535 [M − H − Glc − 2Ac]^−^	195 [M − H − Glc − doBA − Ac_3_Glc]¯	**+**		**+**		**+**		**+**		**+**		**+**		**+**	**+**	T	[6,72]
**29**	Macrophylloside A	Secoiridoid	68.8	234	875	833 [M − H − H_2_O]^−^ 815 [M − H − H_2_O]^−^ 782 739 [M − H − doBA]^−^ 721 713 [M − H − Glc]^−^ 697	619 577 [M − H − Glc − doBA]^−^ 535 [M − H − Glc − doBA − Ac]^−^ 517, 475, 451, 441, 409													**+**		T	[48,72]
**30**	1*α*,2*α*,3*β*,24-tetrahydroxyursa-12,20(30)-dien-28-oic acid	Triterpenoid	72.8	-	501	547 [M − H + HCOOH]^−^ 469, 455, 439, 424, 405, 389															**+**	T	[81]
**31**	Caulophyllogenin	Triterpenoid	75.3	-	487	469 [M − H − H_2_O]^−^ 419															**+**		[82]

#### 2.1.1. Iridoids

Iridoids are a class of monoterpenoid compounds derived from 8-oxogeranial, a key intermediate formed from IPP and DMAPP units supplied predominantly by the plastidial MEP pathway, with possible contributions from the cytosolic MVA pathway [83]. They are typically characterized by a cyclopenta[c]pyran skeleton and frequently occur as glycosides. In plants, iridoids play important physiological roles, including defense against herbivores and pathogens, as well as modulation of growth and development. In the context of medicinal plants, they exhibit a broad spectrum of pharmacological activities, such as anti-inflammatory, hepatoprotective, cardioprotective, and neuroprotective effects [84].

One typical iridoid was identified in the analyzed plant extracts—loganic acid (**7**). Loganic acid is a core iridoid metabolite in the *Gentianaceae* family and is considered a key precursor in the biosynthesis of secoiridoids [85]. The presence of loganic acid has previously been confirmed in both parental species [6,48]. This compound was detected in all shoot samples and, additionally, in the roots of TIB.

#### 2.1.2. Secoiridoids

**Figure 8:**
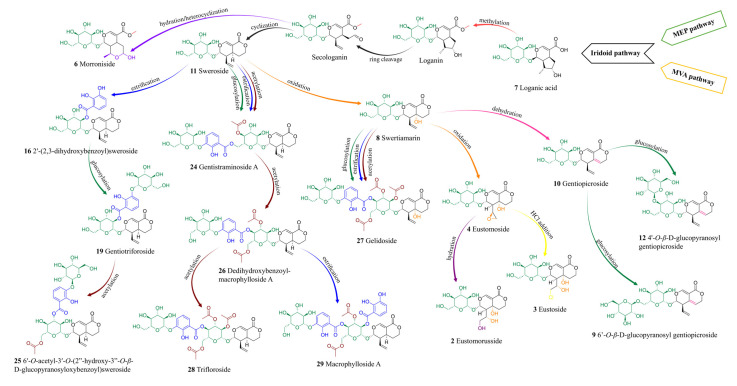
Proposed biosynthetic pathway of identified secoiridoids in somatic hybrids *G. cruciata* (+) *G. tibetica* and parental plants [90].

Secoiridoids are a subclass of monoterpenoids derived from cyclopentane precursors—iridoids—via ring opening between C-7 and C-8 of cyclomethene oxime intermediates [86]. Characterized by a chemically reactive hemiacetal moiety, these compounds exhibit diverse biological activities, including neuroprotective, anti-inflammatory, antidiabetic, hepatoprotective, and analgesic effects. In the *Gentiana* genus, secoiridoids represent one of the most characteristic and pharmacologically important classes of specialized metabolites [86]. Among the analyzed extracts, 19 secoiridoids (including one atypical secoiridoid) were identified that can be arranged in the proposed biosynthesis pathway (Figure 8) beginning with two precursor metabolites: swertiamarin (**8**), from which sweroside (**11**) is subsequently formed. These compounds were consistently detected in the shoots of all tested lines, including the parental plants and all somatic hybrids, and additionally in the roots of *G. tibetica*. Their ubiquitous presence suggests that the early stages of secoiridoid biosynthesis via the MEP, MVA, and secoiridoid pathway [86] remain functionally conserved across both parental and hybrid genotypes.

Notably, gentiopicroside (**10**), derived from the swertiamarin, was identified in all samples regardless of organ type or genotype, as it is the principal secoiridoid in the *Gentiana* genus and a recognized chemotaxonomic marker. Its consistent presence across both parental and hybrid plants confirms that the central branch of the secoiridoid biosynthetic route remains unaffected by the somatic hybridization process. Gentiopicroside is a leading component in the *Gentiana* genus, which possesses a wide spectrum of pharmacological properties, including hepatoprotective, anti-arthritic, analgesic, antidiabetic, anticancer, myorelaxant, anti-aging, and neuropsychopharmacological activities [87]. The stability and abundance of gentiopicroside in parental plants and hybrids highlight its biotechnological relevance for metabolite production in *Gentiana* spp.

The pathway proceeds with further glycosylation and acylation steps, resulting in more specialized compounds. For example, monoglycosylated derivatives of gentiopicroside, namely 6′-O-β-D-glucopyranosylgentiopicroside (**9**) and 4′-O-β-D-glucopyranosylgentiopicroside (**12**), were likewise found in all shoot samples. These compounds likely arise from late-stage glycosylation and reflect a well-maintained enzymatic machinery for sugar conjugation within the aerial tissues. Further along the pathway, the glucosides typical for *G. cruciata* [6] are formed from swertiamarin. Eustomoside (**4**), eustoside (**3**), and eustomorusside (**2**) were present in the shoots of CR but absent in TIB. Eustomoside was detected in all hybrid shoots and eustoside was detected only in F30A-2, while eustomorusside was not detected in any of the hybrids. Compounds from this group provide phytochemical evidence for the functional integration of genes derived from *G. cruciata* and the proper course of their expression.

Gelidoside (also known as rindoside, **27**) is biosynthesized through the conjugation of 2,3-dihydroxybenzoic acid 3-O-β-D-glucopyranoside (**5**), followed by triple acetylation of swertiamarin. This compound was detected in the shoots of all analyzed plants, regardless of their genotype, and was additionally present in the roots of F30A-4 and TIB. Its distribution pattern is consistent with previous reports describing its occurrence in *G. tibetica* [48].

The attachment of a 2,3-dihydroxybenzoyl group to sweroside yields compound **16** (2′-(2,3-dihydroxybenzoyl)sweroside). Subsequent glycosylation results in the formation of gentiotrifloroside (**19**), while an additional acetylation step gives rise to compound **25** (6′-O-acetyl-3′-O-(2″-hydroxy-3″-O-β-D-glucopyranosyloxybenzoyl)-sweroside). Secoiridoids from this biosynthetic branch were detected exclusively in somatic hybrids and *G. tibetica*, indicating that the activation of this pathway is associated with the presence of TIB-derived genetic material.

**Figure 9:**
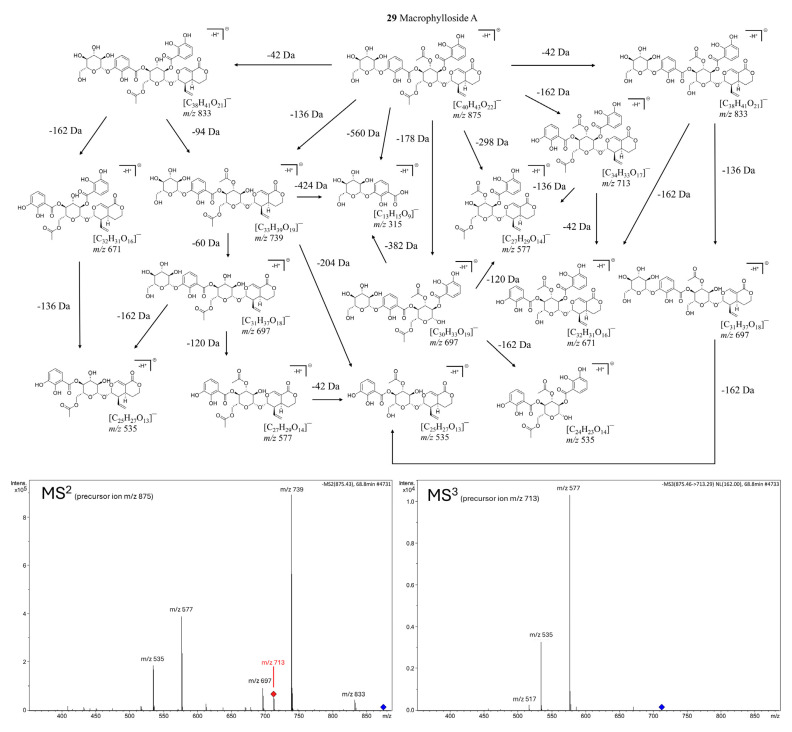
Proposed fragmentation pathway of macrophylloside A in negative ionization mode, based on MS^2^ (precursor ion *m*/*z* 875) and MS^3^ (precursor ion *m*/*z* 713) experiments. The scheme presents deprotonated product ions and their corresponding chemical structures, with indicated neutral losses (Da). Fragmentation steps are corroborated by the most abundant signals in the MSⁿ spectra shown below.

Macrophylloside-type derivatives, including gentistraminoside A (**24**), dehydroxybenzoylmacrophylloside A (**26**), trifloroside (**28**), and macrophylloside A (**29**), were identified in selected lines. The proposed MS/MS fragmentation pathway in negative ion mode for the last one of them elucidates the structures of the product anions illustrated in Figure 9. Their occurrence reflects an expanded biosynthetic capacity leading to higher-order secoiridoid conjugates. These high-molecular-weight compounds highlight the enzymatic plasticity within the genus *Gentiana* and demonstrate the potential of hybrid lines as a novel biosynthetic platform for structurally diverse, functionalized secoiridoids. While acylation may increase the lipophilicity and membrane permeability of metabolites, glycosylation can improve their solubility, stability, and bioavailability, collectively supporting the value of these structural modifications for biotechnological applications. Overall, the somatic hybrids maintained a conserved core profile of secoiridoids (e.g., gentiopicroside, swertiamarin, sweroside) while selectively integrating parental-specific biosynthetic traits. The selection of F30A-5 and F30A-6 hybrids, which combine broad secoiridoid diversity with the production of rare conjugates, presents a promising biotechnological route for targeted metabolite synthesis.

Rare for the gentians, morroniside (**6**) [6] is an atypical secoiridoid with a broken double bond at C-7 and C-8 in the five-membered carbon ring and replaced by a six-membered cyclic inner ether [88]. Although morroniside is often referred to as an iridoid glycoside in the literature, it lacks the intact cyclopenta[c]pyran ring typical of classical iridoids. Structurally, it may be more accurately considered a degraded or secoiridoid-type compound, as it is biosynthetically derived from secologanin [11,89]. This compound was detected exclusively in the shoots of two hybrid lines: F30A-2 and F30A-5. It was not found in any tissues of the parental plants, which may suggest the activation of a previously silent biosynthetic pathway as a result of somatic protoplast fusion. Alternatively, morroniside may be produced in trace amounts by the parental plants, below the detection threshold, but reaches detectable levels in the hybrids due to enhanced biosynthesis.

Additionally, based on comparison with the reference standard, our findings confirm the absence of amarogentin in both parental species and their somatic hybrids. This result is in agreement with previous studies, which have not documented amarogentin in *G. cruciata* or *G. tibetica*, further supporting its chemotaxonomic restriction to other *Gentiana* taxa.

#### 2.1.3. Flavonoids

Flavonoids are widely distributed plant polyphenols that serve essential ecological and physiological functions. In plants, they act as UV-protective pigments, signaling molecules in symbiotic interactions, and defense agents against pathogens and herbivores [91]. In *Gentiana* species, flavonoids, primarily glycosides of flavones, such as luteolin, apigenin, and acacetin, occur in both O- and C-glycosylated forms [6]. These compounds are of considerable pharmacological interest due to their documented antioxidant, anti-inflammatory, and enzyme-inhibitory properties [91]. In the analyzed extracts of parental and hybrid lines, four C-glycosylated flavones were identified: isovitexin (**17**), saponarin (syn. isovitexin-7-O-β-D-glucopyranoside, **13**), isovitexin 4′,7-di-O-β-D-glucopyranoside (**18**), and isoorientin 4′-O-β-D-glucopyranoside (**15**).

**Figure 10:**
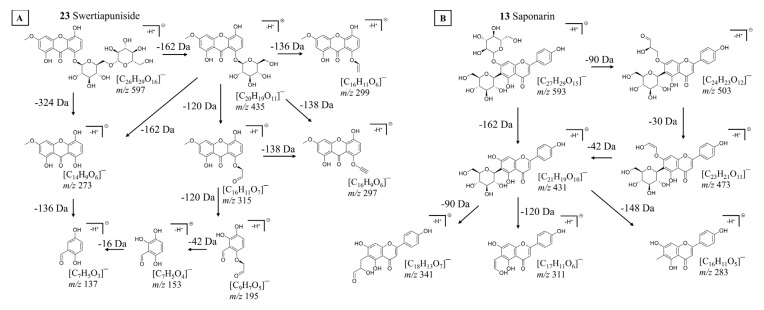
Proposed MS/MS fragmentation pattern in negative ionization mode of deprotonated swertiapuniside (**A**) and saponarin (**B**), and chemical structures of product ions providing main signals in their MS^n^ spectrograms.

Isovitexin was detected exclusively in the shoots of *G. cruciata* and two somatic hybrids (F30A-1 and F30A-2) but was absent in *G. tibetica*. Upon glycosylation at the C-7 position, isovitexin is converted into saponarin, which was consistently present in all plant lines, in both shoot and root tissues. This ubiquitous distribution indicates exceptional biosynthetic stability of this pathway, irrespective of genotype or organ specificity. Given its well-documented antioxidant and anti-inflammatory properties [92], as well as frequent occurrence within the *Gentiana* genus [11], saponarin may serve as a reliable chemotaxonomic flavonoid marker for this group of plants. The proposition of MS/MS fragmentation pattern and chemical structures of product ions detected for deprotonated saponarin is shown in Figure 10B.

The remaining flavonoids exhibited more restricted and genotype-dependent distributions. Isovitexin 4′,7-di-O-β-D-glucopyranoside was detected only in the shoots of CR and the F30A-1 hybrid. In contrast, isoorientin 4′-O-β-D-glucopyranoside was found exclusively in the roots of *G. tibetica* and three hybrid lines (F30A-4, F30A-5, and F30A-6). This pattern suggests a partial inheritance of *G. tibetica*-specific glycosylation traits in selected hybrid genotypes.

#### 2.1.4. Xanthones

Xanthones are oxygenated heterocyclic compounds based on a dibenzo-γ-pyrone scaffold, typically occurring as mono- or polymethyl ethers and glycosides within the *Gentiana* genus [10]. Three xanthones were identified in the analyzed hybrid lines and parental plants: mangiferin (**14**), swertianolin (**22**), and swertiapuniside (**23**). Unlike earlier reports indicating a restricted distribution of xanthones in Gentianaceae [10], all three compounds were consistently detected in the shoot tissues of both parental species and all somatic hybrids, demonstrating not only their stable presence across genotypes but also the robust activity of xanthone biosynthetic pathways. This widespread occurrence highlights the phytochemical value of the generated hybrids and their suitability as uniform sources of these bioactive compounds.

Mangiferin is the most extensively studied xanthone in the *Gentiana* genus, with well-documented anti-inflammatory, antioxidant, neuroprotective, and antidiabetic activities [10,93]. In the present study, mangiferin was detected in the aerial parts of all analyzed genotypes, including both parental species (CR and TIB) and all five somatic hybrids. Notably, this represents the first confirmed occurrence of mangiferin in *G. tibetica*. Its universal presence across genotypes suggests stable and uninterrupted activity of the relevant xanthone biosynthetic pathways, even under in vitro culture conditions and after somatic protoplast fusion.

Similarly, swertianolin and swertiapuniside, glycosylated derivatives of bellidifolin [94], were found in the shoots of all tested genotypes. Interestingly, swertiapuniside was also detected in the roots of hybrids F30A-4, F30A-5, and F30A-6, making it the only xanthone identified in this organ. This tissue-specific shift may indicate partial relocation of the biosynthetic pathway, possibly induced by regulatory changes following somatic hybridization. Such effects could result from novel transcriptional interactions or the activation of a previously silent root-specific biosynthetic route. The proposed MS/MS fragmentation pathway for deprotonated swertiapuniside and chemical structures of product ions providing main signals in its MS^n^ spectrograms are presented in Figure 10A.

#### 2.1.5. Lignans

Lignans are a distinct class of phenylpropanoid dimers, typically formed via β–β′ coupling of two C_6_–C_3_ units [95]. Among all the analyzed extracts, only one lignan was identified—acanthoside B (also known as eleutheroside E1, **20**), a glycosylated derivative of syringaresinol [95]. This compound was exclusively detected in root tissues of both parental species (*G. cruciata* and *G. tibetica*) as well as four somatic hybrid lines (except F30A-4), indicating pronounced organ specificity and a conserved accumulation of this metabolite in underground plant parts. To the best of our knowledge, this is the first report of lignan occurrence in these particular *Gentiana* species.

Acanthoside B is recognized for its antioxidant, immunomodulatory, and anti-inflammatory properties [95]. Its root-specific localization may reflect adaptive significance, as lignans are often implicated in protection against oxidative stress and soil-borne pathogens [96]. Although this compound has rarely been discussed in the context of the *Gentiana* genus, it was previously isolated from *G. purpurea* L. roots [97] and identified in *G. veitchiorum* Hemsl. and *G. szechenyii* Kanitz [68], making its detection in the present study noteworthy and suggestive of a broader lignan profile within this taxon.

#### 2.1.6. Triterpenoids

Triterpenoid structures are formed from six isoprene units, forming a 30-carbon backbone [98]. Triterpenes and triterpenoid saponins are compounds rarely found in plants of the *Gentiana* genus [6]. In the analyzed extracts, two triterpenoids were identified: caulophyllogenin (**31**) and 1α, 2α, 3β, 24-tetrahydroxyurs-12-en-28-oic acid (**30**), the latter of which was first isolated from the flowers of *G. tibetica* by Zhang and Yang [81]. Both compounds were detected exclusively in the roots of *G. tibetica* and were absent from *G. cruciata* and all somatic hybrid lines, indicating high species specificity. Both tetrahydroxyursenoic acid and caulophyllogenin belong to pentacyclic ursane-type triterpenoids, whose presence in *Gentiana* has been reported far less frequently than that of iridoids or secoiridoids [82]. Their restriction to *G. tibetica* suggests that triterpenoid biosynthetic pathways are active only in this parental species and were neither transferred nor activated in the hybrid lines following protoplast fusion.

#### 2.1.7. Other Compounds

In the analyzed extracts, additional compounds were identified that do not belong to the major classes of secondary metabolites discussed above but may nonetheless contribute to the overall biological activity or hold chemotaxonomic significance. This group included one chromene derivative, one phenolic acid compound derivative, and one carbohydrate.

**Figure 11:**
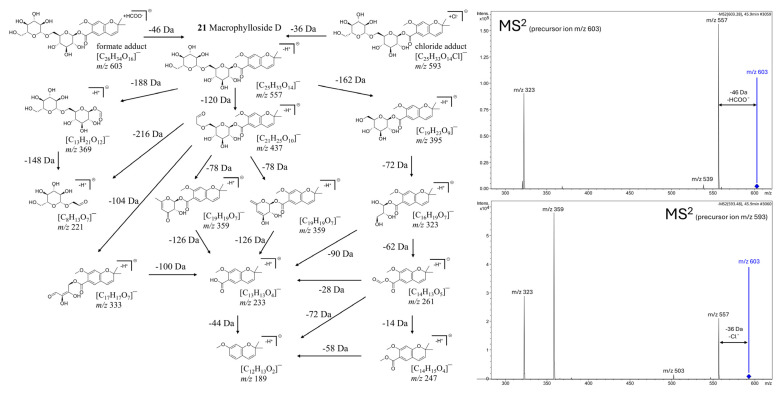
Proposed fragmentation scheme of macrophylloside D in negative ionization mode, supported by MS^2^ spectra of its formate (*m*/*z* 603) and chloride (*m*/*z* 593) adducts shown on the right. The diagram presents deprotonated product ions and their chemical structures, with indicated neutral losses (Da).

Macrophylloside D (**21**), a chromene carboxylic acid derivative glycosyl ester [99,100], emerged as the most abundant peak in BPCs of root extracts across all studied lines, including hybrid roots. Notably, this compound was also detected in the shoots of CR (and the hybrid F30A-4), representing the first report of its occurrence in this species. Until now, macrophylloside D had only been reported in TIB [101]. Furthermore, MS/MS analysis enabled a detailed characterization of macrophylloside D fragmentation, and a proposed scheme of its fragment ions was presented in Figure 11.

2,3-dihydroxybenzoic acid 3-O-β-D-glucopyranoside (**5**), a glycosylated phenolic acid derivative, was present in all shoot samples of the hybrid lines as well as in TIB. This compound serves as a biosynthetic precursor for several complex secoiridoids, such as gelidoside and trifloroside, highlighting its functional importance in the phenolic-acylated branch of secoiridoid biosynthesis.

The identified carbohydrate, gentianose (**1**), a trisaccharide characteristic of the *Gentiana* genus [102], was found exclusively in root samples of all tested plants, except those of *G. tibetica*. Although sugars are classified as primary metabolites, the presence of gentianose may hold chemotaxonomic relevance [102]. Its selective accumulation in root tissues suggests active glycosylation processes and underlines the biosynthetic capacity of roots as a source of glycosidic conjugates.

### 2.2. Evaluation of the TPC, TSC, and Antioxidant Potential Using the DPPH and FRAP Methods

**Figure 12:**
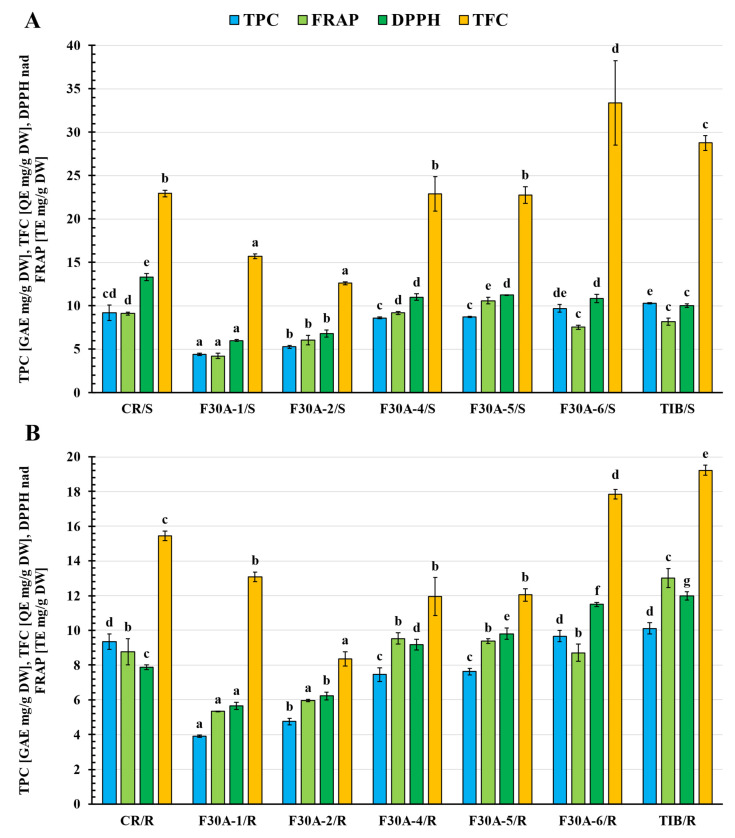
Total phenolic content (TPC) [GAE mg/g DW], total flavonoid content (TFC) [QE mg/g DW], and antioxidant activity determined by the method with DPPH· radical and FRAP method [TE mg/g DW] in shoot (**A**) and root (**B**) extracts of parental plants *G. cruciata* (CR), *G. tibetica* (TIB), and somatic hybrids (F30A-1, F30A-2, F30A-4, F30A-5, and F30A-6). Means followed by the same letter in the columns (individually for shoots and roots extracts) are not significantly different at the level of *p* < 0.05 (HSD Tukey’s test). GAE—gallic acid equivalent; QE—quercetin equivalent; TE—Trolox equivalent.

The total phenolic content (TPC), total flavonoid content (TFC), and antioxidant capacity assessed by FRAP and DPPH assays varied considerably (Figure 12) among the parental species (CR, TIB), somatic hybrids (F30A-1 to F30A-6), and between shoots (S) and roots (R).

Among the parental species, the highest TPC values were recorded in *G. tibetica* shoots (10.28 ± 0.09 mg GAE/g DW) and roots (10.12 ± 0.32 mg GAE/g DW), confirming the strong antioxidant potential of this species. In somatic hybrids, the shoots of line F30A-6 showed the highest TPC (9.69 ± 0.47 mg GAE/g DW), comparable to the parental values. The remaining hybrid shoots showed lower contents, particularly in F30A-1 (4.38 ± 0.13 mg GAE/g DW) and F30A-2 (5.25 ± 0.18 mg GAE/g DW). In general, root tissues of hybrids accumulated lower phenolic levels than the aerial parts, with the exception of F30A-6, where values were nearly identical in both organs (~9.68 mg GAE/g DW).

The highest TFC was observed in the shoots of F30A-6 (33.36 ± 4.89 mg QE/g DW), surpassing both parental genotypes, including *G. tibetica* (28.75 ± 0.83 mg QE/g DW). This suggests a potential transgressive inheritance of flavonoid biosynthetic capacity in this line. Other hybrids, such as F30A-4 (22.88 ± 1.98 mg QE/g DW) and F30A-5 (22.73 ± 0.97 mg QE/g DW), displayed TFC values comparable to those in *G. cruciata* (22.93 ± 0.39 mg QE/g DW), indicating a possible contribution of the CR nuclear background. As expected, TFC levels in roots were markedly lower, with the lowest values observed in F30A-2 (8.36 ± 0.41 mg QE/g DW).

In the FRAP assay, the highest antioxidant capacity was found in the roots of TIB (13.02 ± 0.54 mg TE/g DW). Among hybrids, the most active samples were the shoots of F30A-5 (10.58 ± 0.40 mg TE/g DW) and roots of F30A-4 (9.53 ± 0.34 mg TE/g DW), reaching levels similar to CR shoots (9.10 ± 0.18 mg TE/g DW). The lowest FRAP value was observed in F30A-1 shoots (4.21 ± 0.31 mg TE/g DW), further emphasizing the heterogeneity among hybrid lines.

The DPPH assay revealed the highest antioxidant activity in *G. cruciata* shoots (13.30 ± 0.38 mg TE/g DW). Among the hybrids, the most promising results were observed in the shoots of F30A-5 (11.21 ± 0.02 mg TE/g DW) and roots of F30A-6 (11.50 ± 0.13 mg TE/g DW), both approaching the performance of TIB roots (11.99 ± 0.23 mg TE/g DW). Conversely, the lowest DPPH activity was recorded in F30A-1 roots (5.65 ± 0.22 mg TE/g DW) and shoots (5.99 ± 0.11 mg TE/g DW).

To further explore the relationships between metabolite accumulation and antioxidant capacity, we conducted a Pearson correlation analysis based on data from both parental species and somatic hybrid lines. The analysis was performed separately for shoots and roots to identify organ-specific trends. The results are presented in Figure S1 (Supplementary Materials). Statistically significant correlations (*p* < 0.05) reflect the strength and direction of association between total phenolic content (TPC), total flavonoid content (TFC), and antioxidant activity parameters (FRAP and DPPH), providing insights into how global levels of polyphenols relate to antioxidant potential across genotypes and plant organs. While Pearson correlation analyses were performed across all genotypes (including both parental species and somatic hybrids), it should be noted that such an approach assumes comparable regulatory mechanisms across taxonomically distinct backgrounds. In reality, the biosynthetic relationships between total metabolite content and antioxidant activity may differ between species due to divergence in metabolic regulation, gene expression, or organ-specific compound allocation. Therefore, the observed correlations should be interpreted as reflecting general trends across the tested plant material rather than universal mechanistic rules.

Nonetheless, this integrated correlation-based approach provides valuable and meaningful insights. As shown in Figure S1, the correlation matrix helps identify compound groups that may consistently contribute to antioxidant activity, regardless of genetic background. Notably, strong positive associations were observed between the levels of TPC and antioxidant capacity (FRAP, DPPH) simultaneously in both shoots (r = 0.77 TPC/FRAP and 0.85 TPC/DPPH) and roots (r = 0.84 TPC/FRAP and 0.87 TPC/DPPH, highlighting the central role of phenolic compounds in shaping antioxidant potential across all tested genotypes. Conversely, negative correlations in shoots imply a possible tissue-specific or context-dependent function of these metabolites. Furthermore, calculated r coefficients for FRAP vs. DPPH values (r = 0.87) determined in different samples show convergence of results for both methods and support adequacy of methods chosen for the assessment of antioxidant capacity and reveal sufficiency for the evaluation of the analyzed plant material.

The antioxidant potential and phenolic profiles observed in CR and TIB parental lines were consistent with previous findings reported for *Gentiana* species [6]. In our study, the aerial parts of *G. cruciata* accumulated significantly higher amounts of total flavonoids (22.93 ± 0.39 mg QE/g DW) and exhibited stronger antioxidant activity in both FRAP (9.10 ± 0.18 mg TE/g DW) and DPPH (13.30 ± 0.38 mg TE/g DW) assays compared to roots. This organ-specific pattern aligns with data from Mihailović et al. [49], where methanolic extracts from *G. cruciata* aerial parts displayed over tenfold higher flavonoid content (33.40 ± 1.77 mg RUE/g extract) and approximately twofold higher total phenolic content (59.42 ± 1.62 mg GAE/g extract) than those from roots (GCR). Although these values were expressed per gram of dry extract, the relative differences between plant organs correspond well with our tissue-based data, highlighting the aboveground parts as the main contributors to antioxidant capacity. Interestingly, in TIB, both shoots and roots exhibited comparably high TPC values (~10 mg GAE/g DW), but the highest FRAP activity was found in roots (13.02 ± 0.54 mg TE/g DW), supporting the species’ strong radical scavenging potential. Taken together, our results reinforce literature evidence that phenolic accumulation and antioxidant activity depend on both plant organ and species, while also highlighting the outstanding performance of specific somatic hybrids, which matched their parental lines in these traits.

In summary, the antioxidant profiles of the hybrids demonstrated considerable variability. Lines F30A-5 and F30A-6 exhibited the most favorable antioxidant properties, both in terms of phenolic content and radical scavenging capacity, making them promising candidates for future phytochemical valorization. The observed diversity across hybrid lines highlights the complex inheritance of secondary metabolic traits following somatic hybridization and supports further selection of superior chemotypes.

### 2.3. Quantitative Analysis of Major Iridoids and Secoiridoids in Parental and Somatic Hybrid Gentiana Plants

**Figure 13:**
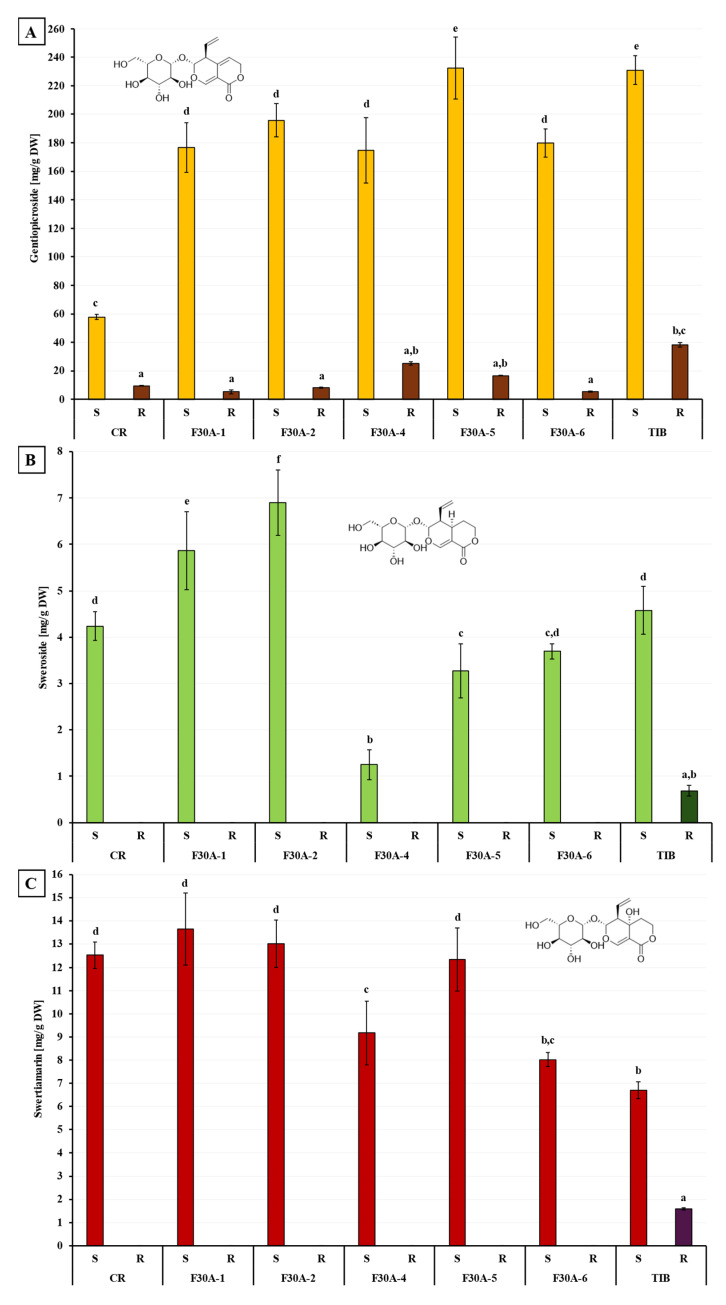
Quantitative analysis of three secoiridoids in shoots (S) and roots (R) of parental plants *G. cruciata* (CR), *G. tibetica* (TIB), and their somatic hybrids (F30A-1, F30A-2, F30A-4, F30A-5, and F30A-6): (**A**) gentiopicroside content; (**B**) sweroside content; (**C**) swertiamarin content. Different lowercase letters indicate statistically significant differences between groups (*p* < 0.05, ANOVA followed by Tukey’s HSD test).

Quantitative UHPLC-DAD analysis revealed significant differences in the levels of gentiopicroside, sweroside, and swertiamarin between the parental species, CR and TIB, and the obtained somatic hybrid lines (F30A-1 to F30A-6), as well as between shoot (S) and root (R) tissues (Figure 13).

Gentiopicroside (Figure 13A) was the quantitatively dominant secoiridoid detected in all plant samples. Among the parental species, TIB shoots exhibited the highest content of gentiopicroside (232.56 ± 10.10 mg/g DW), approximately four times greater than in CR (58.62 ± 1.88 mg/g DW). In the somatic hybrids, gentiopicroside content in shoots ranged from 176.02 ± 22.94 mg/g DW in F30A-4 to 234.20 ± 21.79 mg/g DW in F30A-5. All hybrids showed markedly higher gentiopicroside levels than CR, and one line (F30A-5) reached values comparable to those observed in TIB. In roots, gentiopicroside accumulation was significantly lower, with the highest amounts found in TIB (38.98 ± 1.51 mg/g DW) and F30A-4 (25.80 ± 1.19 mg/g DW), while in the remaining samples, concentrations did not exceed 20 mg/g DW.

For comparison, the gentiopicroside content in *G. cruciata* shoots obtained in this study (58.62 ± 1.88 mg/g DW) fell within the upper range of values previously reported for wild-grown plants (6.3–64.9 mg/g DW) [6,53,103] and was similar to that found in in vitro cultures (49 mg/g DW) as reported by Wójcik and Rybczyński [54]. In *G. tibetica*, the gentiopicroside content (232.56 ± 10.10 mg/g DW) was higher than the levels observed in wild plants (82.3 mg/g DW), in vitro-grown plants (99.6 mg/g DW), and even in genetically modified shoots transformed with *Agrobacterium tumefaciens* strain C58C1 (66.6–124.1 mg/g DW) [54]. In the somatic hybrids, the gentiopicroside content in shoots ranged from 176.02 to 234.20 mg/g DW, reaching values comparable to those in TIB and exceeding those in CR, indicating their high biosynthetic potential. In root tissues, gentiopicroside content in *G. cruciata* (10.11 ± 0.14 mg/g DW) fell within the range reported for in vitro cultures (6.4–12.4 mg/g DW) [38] and was comparable to the maximum levels found in transformed roots obtained with *Agrobacterium rhizogenes* agropine-type A4, 15834, R1000 and mannopine-type 8196 strains by Hayta et al. (up to 10.8 mg/g DW) [21], but substantially lower than those reported for wild roots (28.6–90.5 mg/g DW) and was similar to that found in in vitro cultures (49 mg/g DW) as reported by Wójcik and Rybczyński [54]. In *G. tibetica*, the gentiopicroside content (232.56 ± 10.10 mg/g DW) was higher than the levels observed in wild plants (82.3 mg/g DW), in vitro-grown plants (99.6 mg/g DW), and even in genetically modified shoots transformed with *Agrobacterium tumefaciens* strain C58C1 (66.6–124.1 mg/g DW) [53]. In the somatic hybrids, the gentiopicroside content in shoots ranged from 176.02 to 234.20 mg/g DW, reaching values comparable to those in TIB and exceeding those in CR, indicating their high biosynthetic potential. In root tissues, gentiopicroside content in *G. cruciata* (10.11 ± 0.14 mg/g DW) fell within the range reported for in vitro cultures (6.4–12.4 mg/g DW) [37] and was comparable to the maximum levels found in transformed roots obtained with *Agrobacterium rhizogenes* agropine-type A4, 15834, R1000 and mannopine-type 8196 strains by Hayta et al. (up to 10.8 mg/g DW) [22], but substantially lower than those reported for wild roots (28.6–90.5 mg/g DW) [22,103]. *Gentiana tibetica* roots accumulated higher amounts (38.98 ± 1.51 mg/g DW), while values in hybrids were more variable (5.81–25.80 mg/g DW), falling mostly within the range typical for in vitro plant cultures.

The organ-specific distribution of gentiopicroside observed in this study, with shoots accumulating significantly more than roots, contrasts with patterns reported in wild-grown plants, where root tissues typically serve as the main storage site for this compound. In vitro-grown plants, by contrast, tend to exhibit the opposite pattern, with markedly higher concentrations in aerial parts. This shift in metabolite localization may be attributed to the altered physiology of in vitro cultures, including limited differentiation of root tissues and the juvenile status of explants [104]. In perennial species such as *Gentiana*, roots of mature wild plants can accumulate secondary metabolites over multiple growing seasons, a process that is not replicated in young, short-term in vitro systems [6,103,104]. In vitro-grown plants, by contrast, tend to exhibit the opposite pattern, with markedly higher concentrations in aerial parts. This shift in metabolite localization may be attributed to the altered physiology of in vitro cultures, including limited differentiation of root tissues and the juvenile status of explants [104]. In perennial species such as *Gentiana*, roots of mature wild plants can accumulate secondary metabolites over multiple growing seasons, a process that is not replicated in young, short-term in vitro systems [104].

Sweroside (Figure 13B), a secoiridoid glycoside and biosynthetic intermediate, was primarily detected in shoot tissues. An exception was noted in the roots of TIB, where sweroside was present at a low concentration (0.69 ± 0.11 mg/g DW). Among hybrids, the highest sweroside contents were found in the shoots of F30A-2 (6.90 ± 0.70 mg/g DW) and F30A-1 (5.86 ± 0.84 mg/g DW), which exceeded the levels measured in both parental species (~4.2–4.6 mg/g DW). The lowest sweroside content was observed in line F30A-4 (1.25 ± 0.33 mg/g DW), indicating substantial metabolic variability across the hybrid lines.

Sweroside was present in all shoot samples (CR: 4.24 ± 0.31; TIB: 4.58 ± 0.52; hybrids: 1.25–6.90 mg/g DW), whereas in wild-grown *G. cruciata* its concentration was previously reported to be below the detection limit [6]. This may reflect species-, organ-, or environment-specific regulation of sweroside biosynthesis. In root tissues, sweroside was detected only in *G. tibetica* (0.69 ± 0.11 mg/g DW) and was absent in CR and all hybrid lines (F30A-1 to F30A-6). This observation is consistent with literature data for *G. cruciata* cultivated in vitro, where sweroside levels ranged from undetectable to 0.146 mg/g DW [18]. However, it contrasts with the higher concentration reported by Olennikov et al. in wild *G. cruciata* roots (3.84 ± 0.07 mg/g DW) [6].

Swertiamarin (Figure 13C) was also predominantly accumulated in shoots. High concentrations were observed in CR, F30A-1, F30A-2, and F30A-5 (ranging from 12.33 to 13.66 mg/g DW), while F30A-4, F30A-6, and TIB showed lower values, ranging approximately from 9.2 to 6.7 mg/g DW. In roots, swertiamarin was either undetectable or present in negligible amounts, with the only measurable level recorded in TIB (1.59 ± 0.05 mg/g DW).

Swertiamarin levels in shoots (CR: 12.53 ± 0.57; TIB: 6.70 ± 0.37; hybrids: 8.02–13.66 mg/g DW) were higher than those previously reported for *G. cruciata*, where the compound was below the detection limit [6]. This may indicate activation of swertiamarin biosynthesis under in vitro conditions. In roots, swertiamarin was undetectable in all samples except *G. tibetica* (1.59 ± 0.05 mg/g DW), for which studies of swertiamarin concentration levels were not performed. This finding is noteworthy, as swertiamarin levels previously reported for *G. cruciata* roots ranged from 0.139 to 0.245 mg/g DW under in vitro culture conditions [17] and from 0.306 up to 2.63 mg/g DW in wild-grown plants [6,21].

To deepen the interpretation of phytochemical variation, a targeted correlation analysis was conducted using quantitative data for three major secoiridoids described above, along with corresponding measurements of total phenolic content (TPC), total flavonoid content (TFC), and antioxidant activity (FRAP and DPPH) obtained from the same set of samples. In roots of hybrids and parental species, the concentrations of the three representative secoiridoid compounds exhibited moderate to strong positive correlations with FRAP values, supporting their likely contribution to the overall reductive potential of the extracts.

Additionally, strong positive intercorrelations were observed between the concentrations of the three quantified secoiridoids in root tissues, with the highest values between swertiamarin and sweroside (r = 1.00), and slightly lower yet substantial correlations involving gentiopicroside (r = 0.83). These patterns are consistent with the commonly accepted secoiridoid biosynthetic route in *Gentiana* spp., where sweroside is converted to swertiamarin and subsequently to gentiopicroside, as also depicted in the proposed biosynthetic pathway (Figure 8).

The high secoiridoid content observed in somatic hybrids, particularly for gentiopicroside, may be partially explained by their plastidial inheritance. As shown in our previous study, all hybrid lines retained chloroplasts derived from *G. tibetica* [24], the parent species known to accumulate higher levels of gentiopicroside. Since secoiridoids are synthesized via the plastidial MEP pathway, the retention of TIB plastids may provide a favorable metabolic background for efficient biosynthesis [67]. Moreover, the mitochondrial diversity observed among the hybrid lines, including cases of heteroplasmy, could further contribute to the observed phytochemical variation by modulating energy metabolism or stress-related signaling. Importantly, the somatic hybrids exhibited high genetic stability over long-term culture, as confirmed by molecular marker analysis and the maintenance of distinct nuclear and cytoplasmic profiles across subcultures [24]. Such genetic consistency may support the long-term stability of both qualitative and quantitative aspects of their phytochemical composition, which is particularly important from a biotechnological and standardization perspective.

In summary, the somatic hybrids retained, and in some cases even enhanced, the capacity for secoiridoid biosynthesis, particularly in shoot tissues. Gentiopicroside levels in most hybrids exceeded those found in *G. cruciata* and, in one case, approached the high levels characteristic of *G. tibetica*. Certain lines, such as F30A-2, also exhibited distinct phytochemical traits, including elevated sweroside content. These findings demonstrate that somatic hybridization can generate metabolically diverse lines combining valuable traits of both parental species. However, the altered accumulation patterns observed under in vitro conditions, particularly the pronounced shift of gentiopicroside and other secoiridoids towards shoot tissues, may not fully reflect the biosynthetic potential of mature plants under natural growth regimes. Therefore, to better understand the metabolic stability and agronomic performance of the obtained hybrids, selected lines should be transferred to ex vitro conditions and evaluated in soil-based cultivation. Such studies could determine whether these plants are capable of restoring root-dominant accumulation patterns, as observed in wild populations, and would provide insights into their suitability for long-term phytopharmaceutical applications.

## 3. Materials and Methods

### 3.1. Plant Material

The experiments utilized 5 lines of interspecific somatic hybrids (abbreviated as F30A-1, F30A-2, F30A-4, F30A-5 and F30A-6) obtained following electrofusion of cell suspension-derived protoplasts of *G. cruciata* (Figure 2A) and leaf mesophyll-derived protoplasts of *G. tibetica* (Figure 2B) [47]. Each line was generated from a singular independent post-fusion regenerant (Figure 2C) through clonal propagation via axillary buds. All regenerants were obtained from a single hybrid callus, F30A, through somatic embryogenesis. The F30A-1, F30A-2 and F30A-4 starting plants were regenerated 38 weeks after protoplast fusion, while the F30A-5 and F30A-6 plants were obtained approximately 6 weeks later. The confirmation of the hybrid status of all plants, together with molecular and cytogenetic characteristics, was previously provided by Tomiczak et al. [24]. The reference plant material consisted of *G. cruciata* (Figure 2D) and *G. tibetica* plants (Figure 2E) grown in vitro from seeds collected in the PAS Botanical Garden–CBDC in Powsin, Warsaw, Poland.

All plants (parental and hybrid lines) were cultured in identical glass jars on agar-solidified Murashige & Skoog (MS) medium [105] including vitamins (Duchefa Biochemie B.V., Harleem, The Netherlands; pH=5.8) supplemented with 0.06 M sucrose and maintained in a phytotron at 21 ± 1 °C with a 16-h photoperiod. A photosynthetic photon flux density of 100 μM m^−2^ s^−1^ was provided by daylight fluorescent tubes. Plants were subcultured to a new medium every 6 months. All plants (parental and hybrid lines) were cultured in glass jars on agar-solidified Murashige and Skoog (MS) medium [105]; pH = 5.8, supplemented with 0.06 M sucrose and maintained in a phytotron at 21 ± 1 °C with a 16h photoperiod. A photosynthetic photon flux density of 100 μM m^−2^ s^−1^ was provided by daylight fluorescent tubes. Plants were subcultured to a new medium every 6 months.

### 3.2. Extraction of Plant Material

Phytochemical profiling was performed on shoots (S) and roots (R) of two parental *Gentiana* species, *G. cruciata* (CR) and *G. tibetica* (TIB), as well as five somatic hybrid lines. Both shoots and roots were harvested about 5 months after subculturing. The extraction procedure was adapted from a previously described method [14], with minor modifications. Briefly, freshly collected plant tissues were frozen, lyophilized (Lyophilizator Alpha 2-4 LSC plus, Christ, Osterode am Harz, Germany), and ground into a fine powder. Approximately 100.0 ± 1.0 mg of the dry material was extracted with 30 mL of 100% methanol (Avantor, Gliwice, Poland) using ultrasonic-assisted extraction (Sonorex, Bandelin, Berlin, Germany) for 30 min at 40 °C. The resulting suspensions were centrifuged, and the supernatants were collected. This process was repeated twice. All supernatants were pooled and evaporated to dryness using a rotary evaporator (Rotavapor R-210, Büchi, Flawil, Switzerland) to obtain dry extracts (DEs). Each extraction was performed in triplicate for all plant samples.

### 3.3. Qualitative UHPLC-DAD-ESI-MS^3^ Analysis

The qualitative composition of the obtained extracts was assessed using an ultra-high performance liquid chromatography system coupled with diode array detection and electrospray ionization tandem mass spectrometry (UHPLC-DAD-ESI-MS^3^). The analysis was conducted under conditions adapted from Kiełkiewicz et al. [14], with slight adjustments. Dry extracts (DEs) were reconstituted in 100% methanol with the addition of 0.1% *v*/*v* formic acid to a final concentration of 10.0 mg/mL and filtered through 0.45 µm PVDF syringe filters prior to injection.

Separations were performed on a UHPLC-3000 RS system (Dionex, Dreieich, Germany) equipped with a DAD detector and an AmaZon SL ion trap mass spectrometer (Bruker Daltonik, Bremen, Germany), using an ESI source. Chromatographic separation was achieved on a Kinetex XB-C_18_ column (1.7 µm, 150 × 2.1 mm; Phenomenex, Torrance, CA, USA), maintained at 25 °C. The mobile phase consisted of solvent A (water with 0.1% formic acid, *v*/*v*) and solvent B (acetonitrile with 0.1% formic acid, *v*/*v*). A gradient elution was applied as follows: 0–60 min, 1–26% B; 60–90 min, 26–90% B. The flow rate was 0.3 mL/min, and the injection volume was 5 µL. A 10 min re-equilibration step was performed between injections. UV-Vis spectra were recorded in the range of 200–450 nm. The column effluent was introduced directly into the ESI interface without splitting. The ion source parameters were as follows: nebulizer pressure 38 psi, dry gas (nitrogen) flow 7 L/min, capillary voltage 4.5 kV, and source temperature 135 °C. MS^2^ and MS^3^ fragmentation spectra were collected in Auto MS/MS mode for the most intense precursor ions detected in real time. The scan range was set to *m*/*z* 70–2200 and analyzed in negative ion mode. All solvents used were of HPLC grade (Avantor Performance Materials, Gliwice, Poland).

### 3.4. Metabolite Identification Strategy

In order to identify secondary plant metabolites, a comprehensive literature review was conducted on compounds previously reported in species of the *Gentiana* genus, with particular emphasis on metabolites previously reported in the parental species, *G. cruciata* and *G. tibetica*. Additionally, metabolite structures were searched using the Reaxys and PubChem databases. Most compounds were tentatively identified based on their molecular masses, MS^n^ fragmentation patterns, and UV-Vis absorption spectra, with reference to available literature on *Gentiana* metabolites. For selected compounds, identification was further supported by the use of the following authentic chemical standards: gentiopicroside, sweroside (Toronto Research Chemicals Inc., Canada), swertiamarin (Sigma-Aldrich Chemie, GmbH, Steinheim, Germany), and amarogentin (ChromaDex, Inc., Laguna Hills, CA, USA). The retention times of these standards served as references for assigning identities to other compounds with similar chromatographic and spectral characteristics. In the case of compounds sharing the same molecular mass, the XLogP3-AA lipophilicity values (calculated using XLogP3 3.0, PubChem release 2019.06.18) were also considered as a parameter indicative of elution order. This property was used to support the structural assignment of isomeric or closely related compounds.

### 3.5. Quantitative UHPLC-DAD Analysis

Quantitative analyses of iridoids and secoiridoids were performed based on data acquired using the DAD detector. Dry extracts (DEs) were reconstituted in 100% methanol with the addition of 0.1% *v*/*v* formic acid to a final concentration of 1.0 mg/mL and filtered through 0.45 µm PVDF syringe filters prior to injection. Compound selectivity and identification were supported by previously acquired UHPLC-ESI-MS^3^ spectra to verify retention time and exclude potential co-elution under the applied chromatographic conditions.

Three secoiridoids—gentiopicroside, swertiamarin, and sweroside—were quantified using a commercial reference standard dissolved in 100% methanol (Avantor Performance Materials, Gliwice, Poland). The results were expressed as mg of compound per g of dry weight plant material [mg/g DW]. Absorbance measurements for quantitative analysis were performed at 275 nm for gentiopicroside, 246 nm for sweroside, and 232 nm for swertiamarin, according to Mufasa et al. [106]. Calibration curves were constructed by plotting the peak area against the amount of standard injected. Limits of detection (LOD) and quantification (LOQ) were calculated using the following formulas: LOD = 3.3*SD*/*a* and LOQ = 10*SD*/*a*, where *SD* represents the standard deviation of the response (intercept), and *a* is the slope of the calibration curve, in accordance with ICH Q2(R2) guidelines (ICH, 22 December 2023). Validation parameters for the quantitative method are summarized in Table 2.

### 3.6. Determination of Total Phenolic Content (TPC) and Total Flavonoid Content (TFC)

The total phenolic content (TPC) and total flavonoid content (TFC) of the DEs were determined using spectrophotometric assays, following the procedure described by Pękal and Pyrzynska [107], with minor modifications to microplates according to Kiełkiewicz et al. [14]. Extracts were dissolved in 80% methanol at a final concentration of 10.0 mg/mL and used directly for both assays. All absorbance measurements were performed using an Epoch BioTek microplate reader (Agilent, Santa Clara, CA, USA). Each sample was analyzed in quadruplicate, and results were reported as mean values.

TPC was measured using the Folin–Ciocalteu colorimetric assay, which is based on the reduction of the Folin–Ciocalteu reagent by phenolic compounds to form a blue chromophore. Absorbance was recorded at 765 nm. Results were expressed as milligrams of gallic acid equivalents per gram of dry weight [mg GAE/g DW], based on a standard curve prepared with gallic acid (y = 0.0058x + 0.0247; R^2^ = 0.9980).

TFC was determined using the aluminum chloride (AlCl_3_) colorimetric method in the presence of NaNO_2_ under alkaline conditions. This method is based on the formation of a yellow flavonoid-aluminum complex, with absorbance measured at 425 nm. Results were calculated as milligrams of quercetin equivalents per gram of dry weight [mg QE/g DW], based on a quercetin standard curve (y = 0.0027x + 0.0843; R^2^ = 0.9975).

An Epoch BioTek microplate reader (Agilent, Santa Clara, CA, USA) was used for all assays. Each sample was measured in three replicates, and the mean value was taken.

### 3.7. Determination of Antioxidant Activity (DPPH• and FRAP Assays)

The antioxidant activity of the dry extracts (DEs) was evaluated using the DPPH• radical scavenging assay [108] and the ferric reducing antioxidant power (FRAP) assay [109], with slight modifications to microplates [14]. Extracts were dissolved in 80% methanol at a final concentration of 10.0 mg/mL and used directly for both assays. All absorbance measurements were performed using an Epoch BioTek microplate reader (Agilent, Santa Clara, CA, USA). Each sample was analyzed in triplicate, and the mean value was reported.

The DPPH• (2,2-diphenyl-1-picrylhydrazyl) assay is based on the ability of antioxidant compounds to reduce the stable DPPH• radical to its non-radical form (DPPH-H), resulting in a color change from purple to yellow. Absorbance was measured at 520 nm. A calibration curve was prepared using Trolox as the standard, and results were expressed as milligrams of Trolox equivalents per gram of dry weight [mg TE/g DW], based on the following regression equation: y = 0.0042x − 0.0528; R^2^ = 0.9977.

The FRAP assay measures the ability of antioxidants to reduce ferric ions (Fe^3+^) to ferrous ions (Fe^2+^) in the presence of TPTZ (2,4,6-tripyridyl-s-triazine), forming a blue-colored Fe^2+^-TPTZ complex. The absorbance was measured at 593 nm. Trolox was used to generate the standard curve, and the results were expressed in the same units [mg TE/g DW], using the following calibration equation: y = 0.0081x + 0.0122; R^2^ = 0.9981.

### 3.8. Statistical Analysis

All experimental results are presented as mean values ± standard deviation (SD), based on appropriate biological and analytical replicates. Data were processed using Statistica PL software, version 13 (TIBCO Software Inc., 2017; Palo Alto, CA, USA). Prior to further analyses, the assumptions of normality and homogeneity of variances were evaluated using the Shapiro–Wilk and Brown–Forsythe tests, respectively. For datasets, one-way analysis of variance (ANOVA) followed by Tukey’s Honest Significant Difference (HSD) test was performed to identify statistically significant differences between group means. Groups not significantly different at *p* < 0.05 are denoted by the same letters. To assess chemical similarity among hybrids and parental plants, hierarchical cluster analysis (HCA) was conducted based on a binary presence–absence matrix of identified metabolites. The unweighted pair–group method with arithmetic mean (UPGMA) was applied, using percent disagreement as the distance metric. The Pearson correlation coefficient was employed to express all pairwise correlations between total metabolite content, monitored secoiridoid content, and antioxidant activity. All charts and visualizations were prepared using Microsoft Excel 365 version 2507, build 16.0.19029.20136.

## 4. Conclusions

This study provided a comprehensive molecular and metabolomic characterization of somatic hybrids between two medicinal species, *G. cruciata* and *G. tibetica*. The results revealed that, despite the use of *G. tibetica* mesophyll protoplasts, the hybrid lines exhibited greater genomic and phytochemical similarity to *G. cruciata*. This suggests a hypothesis of the “suspension” fusion partner’s nuclear genome dominant influence on the phenotype of the hybrid’s secondary metabolism.

The high and chemically diverse secondary metabolite profile consisting of secoiridoids, xanthones, flavonoids, iridoids, and lignans observed in somatic hybrids, particularly in lines F30A-5 and F30A-6, underscores their value as elite phytochemical genotypes. Given their confirmed genetic stability over prolonged in vitro cultivation and consistent metabolite accumulation, these hybrids represent promising candidates for scaled-up biotechnological production. One avenue to ensure uniformity and preserve elite traits is clonal propagation via somatic embryogenesis. The feasibility of this approach has been demonstrated for both *G. cruciata* and *G. tibetica*, for which reliable somatic embryogenesis protocols have been developed by Mikuła et al. [51,52]. Application of this method to selected hybrids could enable mass multiplication of phytochemically superior lines and support downstream research on secondary metabolite regulation and production [110]. To secure these unique genotypes for future research or commercial use, cryopreservation may provide an effective long-term storage strategy [111]. Recent work by Markowski et al. [112] on *G. capitata* and *G. decumbens* L.f. confirmed that cryopreservation did not affect DNA content or genetic integrity in these species, indicating that such procedures could be safely applied to conserve valuable hybrid lines without compromising their biotechnological potential.

The observed pattern of gentiopicroside accumulation in shoot tissues in in vitro-grown parental plants and somatic hybrids highlights the influence of culture conditions on secondary metabolite distribution. Further studies should assess the impact of somatic hybridization on the rate of growth in vitro to measure the chosen secondary metabolite yields in optimal culture conditions for biosynthesis efficiency determination. Given their increased chromosomal complement, the hybrids may exhibit enhanced biomass accumulation rates, which could translate into higher productivity of specialized metabolites under specific conditions and timeframes. To fully elucidate the phytochemical potential and stability of the obtained hybrid lines in comparison to the parental species, performance under *ex vitro* or field conditions should also be evaluated. Transferring selected lines to traditional cultivation systems may reveal their long-term capacity for root-based metabolite storage, more closely reflecting natural biosynthetic patterns found in wild *Gentiana* species.

A promising direction for future research would be to investigate the relationship between the parental cell type used for protoplast fusion (suspension-derived vs. mesophyll cells) and the resulting metabolic characteristics of somatic hybrids, particularly after genomic shock, stabilization, and full plant regeneration. Although challenging due to the multistep nature of the hybridization process, such studies could provide valuable insights into the potential for targeted modulation or programming of biosynthetic pathways in somatic hybrids. This could have possible applications in large-scale in vitro production of specialized metabolites without further depletion of natural populations and disturbance of natural habitats.

## Figures and Tables

**Table 2:** Linearity, LOD, and LOQ data for the used standards.

Analyte	λ_det_ [nm]	Calibration Equation	*n*	Correlation Coefficient [R^2^]	Linear Range [ng per injection]	LOD [ng per injection]	LOQ [ng per injection]
Gentiopicroside	275	y = 1.6969x + 27.4931	5	0.9995	100–5000	53.3	159.9
Swertiamarin	232	y = 4.3299x + 2.3544	5	0.9999	5–250	1.1	3.2
Sweroside	246	y = 5.1749x − 1.2439	5	1.0000	5–250	0.8	2.5

λ_det_—detection wavelength used during quantification; y—peak area; x—the amount of compound injected onto the column; *n*—number of concentration levels for the calibration curve; LOD—limit of detection; LOQ—limit of quantification; R^2^—correlation coefficient for the linear model.

## Data Availability

The dataset is available on request from the authors.

## Supplementary Materials

The following supporting information can be downloaded at https://www.mdpi.com/article/10.3390/molecules30163321/s1, Table S1: List of all detected metabolites in methanolic extracts from shoots (S) and roots (R) of parental species (*G. cruciata*—CR and *G. tibetica*—TIB) and their somatic hybrids (F30A-1 to F30A-6), obtained using UHPLC-DAD-ESI-MS^3^ analysis. Figure S1: Correlation matrix illustrating relationships between total phenolic content (TPC), total flavonoid content (TFC), antioxidant activity parameters (FRAP, DPPH), and concentrations of selected representative metabolites (gentiopicroside, swertiamarin, sweroside) detected in shoots and roots of somatic hybrids and parental species (*G. cruciata* and *G. tibetica*). Pearson correlation coefficients statistically significant at *p* < 0.05 are marked with color: green—denoting strong positive correlation, and red—denoting strong negative correlation.

## Abbreviations

The following abbreviations are used in this manuscript:

**Ac**	acetyl
**BPC**	base peak chromatogram
**CR**	*Gentiana cruciata*
**CMS**	cytoplasmic male sterility
**DAD**	diode array detector
**DE**	dry extract
**DMAPP**	dimethylallyl pyrophosphate
**doBA**	dihydroxybenzoic acid
**DW**	dry weight
**ESI**	electrospray ionization
**F30A-1/2/4/5/6**	somatic hybrid lines of *G. cruciata* (+) *G. tibetica*
**Frc**	fructose
**GAE**	gallic acid equivalent
**Glc**	glucose
**IPP**	isopentenyl pyrophosphate
**MEP**	methylerythritol phosphate pathway (plastidial)
**MS**	mass spectrometry
**MVA**	mevalonic acid pathway (cytosolic)
**S**	shoots
**R**	roots
**TFC**	total flavonoid content
**TPC**	total phenolic content
**TE**	Trolox equivalent
**TIB**	*Gentiana tibetica*
**UHPLC**	ultra-high-performance liquid chromatography
**UPGMA**	unweighted pair group method with arithmetic mean
**UV-Vis**	ultraviolet-visible spectroscopy
**[M–H]^−^**	deprotonated pseudomolecular ion in negative mode
