# Supplementary material for: Defining Phytochemical Metabolomes of Somatic Hybrids Gentiana cruciata L. (+) G. tibetica King ex Hook.f. (Gentianaceae) Using UHPLC-DAD-ESI-MS3 Analysis in Comparison to the Parental Species
Source: Molecules. 2025 Aug 8;30(16):3321. doi: 10.3390/molecules30163321 (PMC12388807; doi:10.3390/molecules30163321)
Supplement: Supplementary file 1 [file molecules-30-03321-s001.zip › molecules-3797718-supplementary.pdf]

*Article*

# Defining phytochemical metabolomes of somatic hybrids *Gentiana cruciata* L. (+) *G. tibetica* King ex Hook.f. (Gentianaceae) using UHPLC-DAD-ESI-MS³ analysis in comparison to the parental species

**Maciej Obrębski [1,*], Rafał M. Kiełkiewicz [2,*], Karolina Tomiczak [3,**] and Anita A. Śliwińska [2]**

[1] MicrobiotaLab, Department of Pharmaceutical Microbiology and Bioanalysis, Faculty of Pharmacy, Medical University of Warsaw, Banacha 1, 02-097 Warsaw, Poland
[2] Department of Pharmaceutical Biology, Faculty of Pharmacy, Medical University of Warsaw, Banacha 1, 02-097 Warsaw, Poland
[3] Plant Breeding and Acclimatization Institute—National Research Institute in Radzików, Radzików, 05-870 Błonie, Poland

* These authors contributed equally
** Correspondence: k.tomiczak@ihar.edu.pl (K. Tomiczak), Plant Breeding and Acclimatization Institute—National Research Institute in Radzików, Radzików, 05-870 Błonie, Poland, Tel.: +48227334643

**Table S1**. List of all detected metabolites in methanolic extracts from shoots (S) and roots (R) of parental species (*G. cruciata* – CR and *G. tibetica* – TIB) and their somatic hybrids (F30A-1 to F30A-6), obtained using UHPLC-DAD-ESI-MS³ analysis.

The table lists retention times ($t_R$), UV-Vis absorption maxima, deprotonated pseudomolecular ions ($[M–H]^-$), major MS² and MS³ fragment ions, as well as compound occurrence (+) across the analyzed samples. The column marked "C/T" indicates whether a given compound has been previously reported in *G. cruciata* (C) and/or *G. tibetica* (T) according to literature sources.

| No | Compound | Metabolite group | $t_R$ [min] | UV-Vis [nm] | $[M-H]^-$ *m/z* | MS² ions *m/z* | MS³ ions *m/z* | CR S | CR R | F30A-1 S | F30A-1 R | F30A-2 S | F30A-2 R | F30A-4 S | F30A-4 R | F30A-5 S | F30A-5 R | F30A-6 S | F30A-6 R | TIB S | TIB R | Previously reported in C/T | Ref. |
|---|---|---|---|---|---|---|---|---|---|---|---|---|---|---|---|---|---|---|---|---|---|---|---|
| | | | 1.5 | 190 | 719 | 377 | | + | + | + | + | + | + | + | + | | + | + | + | + | | | |
| | | | 1.7 | 192 | 439 | 421, 379, 341, 323, 277, 259, 215, 179 | | + | | + | | + | + | + | + | + | + | + | + | + | + | | |
| | | | 1.9 | - | 637 | 504 | | + | | | | | | | | | | | | | | | |
| 1 | Gentianose | Carbohydrates | 2.0 | - | 503 | 549 $[M-H+HCOOH]^-$<br>341 $[M-H-Frc]^-$<br>179 $[M-H-Frc-Glc]^-$ | | | + | | + | | + | | + | | + | | + | | | C | [1] |
| 2 | Eustomorusside | Secoiridoid | 3.1 | - | 407 | 453 $[M-H+HCOOH]^-$<br>329<br>245 $[M-H-Glc]^-$<br>209 $[M-H-Glc-H_2O-H_2O]^-$ | | + | | | | | | | | | | | | | | | [2] |
| | | | 4.7 | 206 | 479 | 407, 329, 209 | | + | | | | | | | | | | | | | | | |
| | | | 6.9 | 199 | 231 | 213, 169, 139 | | | | | | | | + | | | | | | + | | | |
| | | | 8.1 | 198, 208 | 339 | 385, 207 | | | + | | | | + | | | | | | | | | | |
| | | | 9.1 | 212 | 523 | 407, 329, 209 | | + | | | | | | | | | | | | | | | |

| | | | | | | | | | | | | | | | | | | | | | | |
|---|---|---|---|---|---|---|---|---|---|---|---|---|---|---|---|---|---|---|---|---|---|---|
| | | | 9.9 | 218 | 523 | 407, 329, 209 | + | | | | | | | | | | | | | | | |
| | | | 11.7 | 218 | 565 | 517 | + | | | | | | | | | | | | | | | |
| | | | 12.7 | 215<br>195 | 353 | 399 | | + | | | | | | | | | | | | | | |
| | | | 12.9 | 215<br>195 | 353 | 399 | | | | | | | | | | | | | | + | | |
| **3** | Eustoside | Secoiridoid | 13.0 | - | 425 | 471 [M – H + HCOOH]$^-$<br>263 [M – H – Glc]$^-$<br>208, 141 | + | | | | + | | | | | | | | | | | [3] |
| **4** | Eustomoside | Secoiridoid | 13.8 | - | 389 | 435 [M – H + HCOOH]$^-$<br>227 [M – H – Glc]$^-$<br>209 [M – H – Glc – $H_2O$]$^-$<br>141 | + | | + | | + | | + | | + | | + | | | | C | [3, 4] |
| **5** | 2,3-dihydroxybenzoic acid 3-*O*-*β*-D-glucopyranoside | Phenolic acid glycoside | 14.3 | 308 | 315 | 153 [M – H – Glc]$^-$ | | | + | | + | | + | | + | | + | | + | | | [5] |
| | | | 14.6 | 215 | 427.3 | 397, 383, 381, 331 | | | | | | | | | | | | | | + | | |
| | | | 16.1 | 224 | 449 | 495, 323 | | + | | + | | + | | | | | | | | | | |
| | | | 16.2 | 225 | 405.8 | 179 | | | | | | | | | | | | | | + | | |
| | | | 16.4 | 225<br>215 | 407.4 | 377, 361, 343, 253 | | | | | | | | | | | | | | + | | |
| | | | 17.6 | 226 | 359 | 405, 225, 179 | | | | | | | | | | | | | | + | | |
| | | | 19.6 | 229 | 445 | 491, 359, 323, 179 | + | + | + | | | | + | | | | + | | + | + | | |
| | | | 19.8 | 226 | 361 | 407, 179 | | | | | | | | | | | | | | + | | |
| **6** | Morroniside | Iridoid | 20.1 | - | 405 | 451 [M – H + HCOOH]$^-$<br>243 [M – H – Glc]$^-$ | | | + | | + | | | | | | | | | | | [3, 6] |
| **7** | Loganic acid | Iridoid | 20.3 | - | 375 | 323<br>213 [M – H – Glc]$^-$<br>169 [M – H – Glc – $CO_2$]$^-$ | + | | + | | + | | + | | + | | + | | + | + | C,T | [3, 7] |
| | | | 20.8 | 226 | 419 | 465, 293 | | + | | + | | + | | | | | | | | | | |
| **8** | Swertiamarin | Secoiridoid | 23.0 | - | 373 | 419 [M – H + HCOOH]$^-$<br>211 [M – H – Glc]$^-$<br>179 [Glc – H]$^-$<br>161 [Glc – H – $H_2O$]$^-$ | + | | + | | + | | + | | + | | + | | + | + | C,T | [3, 7, 8] |
| **9** | 6'-*O*-*β*-D-glucopyranosyl gentiopicroside | Secoiridoid | 24.1 | - | 517 | 563 [M – H + HCOOH]$^-$<br>341 [2Glc + $H_2O$ – H]$^-$<br>323 [2Glc – H]$^-$<br>281, 251<br>193 [M – H – 2Glc]$^-$<br>179 [Glc – H]$^-$ | + | | + | | + | | + | | + | | + | | + | + | C,T | [6, 9] |
| | | | 24.6 | 215 | 387 | 433, 179 | | | | | + | | | | + | | | | | | | |
| | | | 24.8 | 229 | 405 | 451, 273 | | | | | | | | + | | + | | + | | | | |
| | | | 25.1 | 233 | 415 | 461, 293 | | + | | | | | | + | | | | | | + | | |
| **10** | Gentiopicroside (syn. gentiopicrin) | Secoiridoid | 25.3 | 274<br>243 | 355 | 401 [M – H + HCOOH]$^-$<br>225<br>193 [M – H – Glc]$^-$<br>179 [Glc – H]$^-$<br>143 [Glc – H – $2H_2O$]$^-$<br>119 [Glc – H – $(CHOH)_2$]$^-$ | + | + | + | + | + | + | + | + | + | + | + | + | + | + | C,T | [3, 7, 8, 10] |
| | | | 25.9 | 227 | 445 | 431, 311, 269 | | + | | | | | | | | | | | | | | |
| **11** | Sweroside | Secoiridoid | 26.6 | 244 | 357 | 403 [M – H + HCOOH]$^-$<br>267 [M – H – $(CHOH)_3$]$^-$<br>195 [M – H – Glc]$^-$<br>179 [Glc – H]$^-$ | + | | + | | + | | + | | + | | + | | + | + | C,T | [3, 7, 8] |

| | | | | | | | | | | | | | | | | | | | | | | | |
|---|---|---|---|---|---|---|---|---|---|---|---|---|---|---|---|---|---|---|---|---|---|---|---|
| | | | | | | 161 $[Glc - H - H_2O]^-$<br>125 | | | | | | | | | | | | | | | | | |
| **12** | 4'-*O*-$\beta$-D-glucopyranosyl gentiopicroside | Secoiridoid | 27.5 | - | 517 | 563 $[M - H + HCOOH]^-$<br>355 $[M - H - Glc]^-$<br>341 $[2Glc + H_2O - H]^-$<br>179 $[Glc - H]^-$ | | + | | + | | + | | + | | + | | + | | + | | | [9] |
| | | | 28.4 | 229 | 329 | 167 | | | | | | | | | | | | | | + | | | |
| | | | 28.4 | 228 | 345 | 391 | | | | | | | | | | | | | | | + | | |
| **13** | Saponarin (syn. isovitexin-7-*O*-$\beta$-D-glucopyranoside) | Flavonoid | 28.8 | 214, 270 | 593 | 503 $[M - H - (CHOH)_3]^-$<br>473 $[M - H - (CHOH)_4]^-$<br>431 $[M - H - Glc]^-$<br>341 $[M - H - Glc - (CHOH)_3]^-$<br>311 $[M - H - Glc - (CHOH)_4]^-$<br>283 | | + | + | + | + | + | + | + | + | + | + | + | + | + | + | C,T | [3, 6] |
| **14** | Mangiferin | Xanthone | 29.1 | 257, 319 | 421 | 403 $[M - H - H_2O]^-$<br>331 $[M - H - (CHOH)_3]^-$<br>301 $[M - H - (CHOH)_4]^-$<br>259 $[M - H - Glc]^-$ | | + | | + | | + | | + | | + | | + | | + | | C | [3, 6-8] |
| **15** | Isoorientin 4'-*O*-$\beta$-D-glucopyranoside | Flavonoid | 29.2 | 227 | 609 | 447 $[M - H - Glc]^-$<br>357 $[M - H - Glc - (CHOH)_3]^-$<br>327 $[M - H - Glc - (CHOH)_4]^-$<br>299 | | | + | | | | | | + | | + | | + | | | C | [3, 6] |
| | | | 29.4 | 218 | 387 | 433 | | | | | | | + | | | | | | | | | | |
| | | | 30.3 | 230 | 599 | 299, 211 | | | | + | | | | + | | + | | | | + | + | | |
| | | | 30.5 | 229 | 387 | 433 | | | + | | | | | | | | | | | | | | |
| | | | 31.1 | 229 | 623.3 | 503, 341 | | | + | | | | | | | | | | | | | | |
| **16** | 2'-(2,3-dihydroxybenzoyl)sweroside | Secoiridoid | 31.9 | - | 493 | 539 $[M - H + HCOOH]^-$<br>339 $[M - H - doBA - H_2O]^-$<br>179 $[Glc - H]^-$ | | | | | | | | + | + | | + | | + | | + | T | [11] |
| | | | 33.9 | 230 | 509 | 555, 347 | | | | | | | | | | | | | | | + | | |
| | | | 35.7 | 230 | 495 | 541, 333, 161 | | | | | | | | | | + | | | | | | | |
| | | | 37.5 | 220 | 415 | 461, 397, 221, 161 | | | | + | | + | | + | | + | | + | | | | | |
| | | | 38.5 | 217 | 639 | 477, 315 | | | | + | | | | + | | | | | | + | | | |
| **17** | Isovitexin | Flavonoid | 39.9 | 230 | 431 | 413 $[M - H - H_2O]^-$<br>341 $[M - H - (CHOH)_3]^-$<br>311 $[M - H - (CHOH)_4]^-$<br>283 | | + | | + | | + | | | | | | | | | | C,T | [3] |
| | | | 40.1 | 220 | 497 | 543 | | | | + | | + | | | | + | | | | | | | |
| | | | 40.7 | 232 | 635 | 617, 427 | | | | | | | | | + | | | | | + | | | |
| | | | 41.4 | 231 | 719 | 765, 485, 383 | | | | | | | | | | | | | + | | | | |
| **18** | Isovitexin 4',7-di-*O*-$\beta$-D-glucopyranoside (syn. saponarin 4'-*O*-$\beta$-D-glucopyranoside) | Flavonoid | 42.4 | 216 | 755 | 593 $[M - H - Glc]^-$<br>431 $[M - H - 2Glc]^-$ | 311 $[M - H - 2Glc - (CHOH)_4]^-$ | + | | + | | | | | | | | | | | | C | [8] |
| | | | 43.3 | 218 | 399 | 445, 194 | | | | + | | + | | | | | | | | | | | |
| | | | 44 | 232 | 719 | 765, 485 | | | | | | | | | | | + | | + | | | | |
| **19** | Gentiotrifloroside (syn. 2-*O*-(2-hydroxy-3-*O*-$\beta$-D-glucopyranosyl-oxybenzoyl)sweroside) | Secoiridoid | 44.1 | 220 | 655 | 493 $[M - H - Glc]^-$<br>195 $[M - H - Glc - doBA - Glc]^-$ | | | | + | | + | | | | + | | | | | | | [6] |
| **20** | Acanthoside B (syn. eleutheroside E1; syringaresinol 4-*O*-$\beta$-D-glucopyranoside) | Lignan | 44.6 | 231 | 579 | 417 $[M - H - Glc]^-$<br>181, 166 | | | + | | + | | + | | | | + | | + | | + | | [1] |
| | | | 45.9 | 219 | 523 | 569, 241 | | | | + | | + | | | | + | | + | | | | | |

| | | | | | | | | | | | | | | | | | | | | | | | |
|---|---|---|---|---|---|---|---|---|---|---|---|---|---|---|---|---|---|---|---|---|---|---|---|
| **21** | Macrophylloside D | Iridoid | 45.9 | 242,<br>324 | 557 | 03 [M – H + HCOOH]$^-$<br>593 [M – H + HCl]$^-$<br>437, 369<br>359 [M – H – Glc – 2$H_2O$]$^-$<br>323, 263, 247,<br>233 [M – H – 2Glc]$^-$<br>221<br>179 [Glc – H]$^-$ | | + | + | | + | | + | + | + | | + | | + | | + | T | [12] |
| | | | 46.2 | 217 | 769 | 607 | 413, 293 | + | | + | | + | | | | + | | + | | | | | |
| | | | 46.4 | 232 | 527 | 573, 323 | | | | | | | | | | | | | | | + | | |
| | | | 46.7 | | 647 | 503, 323, 221 | | | + | | | | | | | | | | | | | | |
| | | | 47 | 235 | 527 | 573, 323 | | | + | | + | | + | | + | | + | | + | | + | | |
| **22** | Swertianolin | Xanthone | 47.9 | 210,<br>310 | 435 | 417 [M – H – $H_2O$]$^-$<br>315 [M – H – $(CHOH)_3$]$^-$<br>287<br>273 [M – H – Glc]$^-$<br>197, 187, 137 | 229 [M – H – Glc – $CO_2$]$^-$<br>185 [M – H – Glc – 2$CO_2$]$^-$ | + | | + | | + | | + | | + | | + | | + | | | [13] |
| | | | 48 | 232 | 557 | 603, 323, 179 | | | + | | | | | | | | + | | + | | | | |
| **23** | Swetiapuniside | Xanthone | 48.5 | 233 | 597 | 435 [M – H – Glc]$^-$ | 315, 299, 297<br>273 [M – H – 2Glc]$^-$<br>229 [M – H – 2Glc – $CO_2$]$^-$<br>195, 153, 137 | + | | + | | + | | + | + | + | + | + | + | + | | | [14] |
| | | | 48.5 | 211 | 527 | 573, 293 | | | + | | + | | + | | | | | | | | | | |
| | | | 48.8 | 220 | 441 | 315, 153 | | | | + | | + | | | | + | | | | + | | | |
| | | | 48.8 | 231 | 683 | 477 | | | + | | | | | | | | | | | | | | |
| | | | 50.5 | 231 | 497 | 543, 293 | | | + | | | | | | | | | | | | | | |
| **24** | Gentistraminoside A (syn. 4’-*O*-acetyl-6’-*O*-(2”-hydroxy-3”-*O*-*β*-D-glucopyranosyloxybenzoyl)-sweroside) | Secoiridoid | 50.8 | 219 | 697 | 655 [M – H – Ac]$^-$<br>571<br>535 [M – H – Glc]$^-$<br>409 | 475 [M – H – Glc – Ac – $H_2O$]$^-$<br>357 [M – H – Glc – doBA – Ac]$^-$<br>349, 153 | | | | | + | | | | | | | | + | | | [6, 15] |
| | | | 51 | 232 | 487 | 441, 143 | | | | | | | | | | | | | | + | | | |
| **25** | 6′-*O*-acetyl-3′-O-(2”-hydroxy-3”-*O*-*β*-D-glucopyranosyloxybenzoyl)-sweroside | Secoiridoid | 51.1 | 231 | 697 | 743 [M – H + HCOOH]$^-$<br>535 [M – H – Glc]$^-$ | | | | | | | | | | | | | | | + | | [3, 6] |
| | | | 51.6 | 232 | 451.3 | 315 | | | | | | | | | | | | | | + | | | |
| | | | 52.1 | 233 | 395 | 441, 233 | | | | | | | | | + | | + | | + | | + | | |
| | | | 55.2 | 232 | 525 | 571, 323 | | | | | | | | | | | | | | | + | | |
| | | | 57.2 | 232 | 545 | 503, 499, 253 | | + | | | | | | | | | | | | + | | | |
| **26** | Dedihydroxybenzoate-macrophylloside A | Secoiridoid | 59.8 | 232 | 739 | 697 [M – H – Ac]$^-$<br>577 [M – H – Glc]$^-$<br>535 [M – H – Glc - Ac]$^-$ | 451 | | | | | | | | | | | | | + | | | [6] |
| | | | 60 | 219 | 607 | 413 | | | | | | + | | | | | | | | | | | |
| | | | 62.4 | 233 | 529 | 453, 227 | | | | | | | | | | | | | | + | | | |
| **27** | Gelidoside (syn. rindoside) | Secoiridoid | 66.6 | 233 | 797 | 755 [M – H – Ac]$^-$<br>713 [M – H – 2Ac]$^-$<br>635 [M – H – Glc]$^-$ | 593 [M – H – Glc – Ac]$^-$<br>373 [M – H – Glc – doBA – 3Ac]$^-$ | + | | + | | + | | + | + | + | | + | | + | + | C,T | [3, 6, 15, 16] |
| | | | 67.6 | 233 | 781 | 827, 763, 587, 485, 383, 341 | | + | | + | | + | | | | | | | | + | | | |
| **28** | Trifloroside | Secoiridoid | 68.4 | 237 | 781 | 739 [M – H – Ac]$^-$<br>619 [M – H – Glc]$^-$<br>577 [M – H – Glc – Ac]$^-$<br>535 [M – H – Glc – 2Ac]$^-$ | 195 [M – H – Glc – doBA – $Ac_3Glc$]$^-$ | + | | + | | + | | + | | + | | + | | + | + | C,T | [3, 6] |
| **29** | Macrophylloside A | Secoiridoid | 68.8 | 234 | 875 | 833 [M – H – $H_2O$]$^-$<br>815 [M – H – $H_2O$]$^-$<br>782<br>739 [M – H – doBA]$^-$ | 619<br>577 [M – H – Glc – doBA]$^-$<br>535 [M – H – Glc – doBA – Ac]$^-$<br>517, 475, 451, 441, 409 | | | | | | | | | | | | | + | | T | [6, 11] |

| | | | | | | | | | | | | | | | | | | | | | | |
|---|---|---|---|---|---|---|---|---|---|---|---|---|---|---|---|---|---|---|---|---|---|---|
| | | | | | | 721<br>713 [M – H – Glc]$^-$<br>697 | | | | | | | | | | | | | | | | |
| | | | 72 | 222 | 341 | 387 | | | | + | | | | | | | | | | | | |
| **30** | 1$\alpha$,2$\alpha$,3$\beta$,24-tetrahydroxyursa-12,20(30)-dien-28-oic acid | Triterpenoid | 72.8 | - | 501 | 547 [M – H + HCOOH]$^-$<br>469, 455, 439, 424, 405, 389 | | | | | | | | | | | | | | + | T | [17] |
| | | | 73.9 | - | 503 | 549 | | + | | + | | + | + | + | | + | | + | | + | | |
| | | | 74.4 | - | 717 | 657 | + | + | + | | + | + | + | | + | | + | | | | | [18] |
| **31** | Caulophyllogenin | Triterpenoid | 75.3 | - | 487 | 469 [M – H – $H_2O$]$^-$<br>419 | | | | | | | | | | | | | | + | | |
| | | | 79 | - | 565 | 504 | | + | | | | | | | | | | | | | | |
| | | | 79.2 | - | 513 | 559, 277 | + | | + | | + | | | | + | | | | | | | |
| | | | 81.5 | - | 471 | 407, 383 | | | | | | | | | | | | | | + | | |
| | | | 85.1 | - | 265 | 97 | + | + | | + | | + | | | + | + | | | | | | |
| | | | 85.3 | - | 513 | 445, 284 | | | | | + | | | | | | | | | | | |
| | | | 87.5 | -- | 309 | 185 | | | | + | | | | | | + | | | | | | |
| | | | 87.7 | - | 310 | 97 | | | | | | | | | + | | | | | | | |
| | | | 87.7 | - | 617 | 497 | | | | | + | | | | | | | | | | | |
| | | | 88.5 | - | 353 | | | | | + | | | | | | | | | | | | |
| | | | 88.7 | - | 822 | 762, 513, 279 | + | | | | | | + | | + | | + | | + | | | |
| | | | 89.1 | - | 397 | | | | | + | | | | | | | | | | | | |
| | | | 89.2 | - | 822 | 762, 513, 279 | + | | | | | | + | | + | | + | | + | | | |
| | | | 89.4 | - | 441 | | | | | + | | | | | | | | | | | | |
| | | | 89.6 | - | 376 | 356 | | | | | | + | + | | | | | | | | | |
| | | | 89.6 | - | 485 | | | | | + | | | | | | | | | | | | |
| | | | 89.8 | - | 409 | 455, 393 | | | | | | | | | | | | | + | | | |
| | | | 90 | - | 914 | 960, 658, 473 | | | | | + | | | | | | | | | | | |


1. Li, Y.; Zhang, J.; Zhang, J.-l.; Fan, J.-y.; Zhao, Q.; Chu, Q.-q.; Zhong, S.-h.; Gu, R., Comprehensive comparison on antioxidant properties and UPLC-Q-TOF/MS-based metabolomics discrimination between *Gentiana veitchiorum* and *G. szechenyi*i. *Arabian Journal of Chemistry* **2024,** 17, (4).
2. Pan, Z.; Xiong, F.; Chen, Y. L.; Wan, G. G.; Zhang, Y.; Chen, Z. W.; Cao, W. F.; Zhou, G. Y., Traceability of Geographical Origin in *Gentiana straminea* by UPLC-Q Exactive Mass and Multivariate Analyses. *Molecules* **2019,** 24, (24).
3. Olennikov, D. N.; Gadimli, A. I.; Isaev, J. I.; Kashchenko, N. I.; Prokopyev, A. S.; Kataeva, T. N.; Chirikova, N. K.; Vennos, C., Caucasian Gentiana Species: Untargeted LC-MS Metabolic Profiling, Antioxidant and Digestive Enzyme Inhibiting Activity of Six Plants. *Metabolites* **2019,** 9, (11).
4. Uesato, S.; Hashimoto, T.; Inouye, H., Three new secoiridoid glucosides from *Eustoma russellianum*. *Phytochemistry* **1979,** 18, (12), 1981-1986.
5. Pan, Y.; Zhang, J.; Zhao, Y. L.; Wang, Y. Z.; Huang, H. Y., Investigation of metabolites accumulation in medical plant *Gentiana rigescens* during different growing stage using LC-MS/MS and FT-IR. *Bot Stud* **2015,** 56, (1), 14.
6. Zhang, G.; Li, Y.; Wei, W.; Li, J.; Li, H.; Huang, Y.; Guo, D. A., Metabolomics Combined with Multivariate Statistical Analysis for Screening of Chemical Markers between *Gentiana scabra* and *Gentiana rigescens*. *Molecules* **2020,** 25, (5).
7. Pan, Y.; Zhang, J.; Shen, T.; Zhao, Y. L.; Wang, Y. Z.; Li, W. Y., Comparative metabolic fingerprinting of *Gentiana rhodantha* from different geographical origins using LC-UV-MS/MS and multivariate statistical analysis. *BMC Biochem* **2015,** 16, 9.
8. Mihailović, V.; Mišić, D.; Matić, S.; Mihailović, M.; Stanić, S.; Vrvić, M. M.; Katanić, J.; Mladenović, M.; Stanković, N.; Boroja, T.; Stanković, M. S., Comparative phytochemical analysis of *Gentiana cruciata* L. roots and aerial parts, and their biological activities. *Industrial Crops and Products* **2015,** 73, 49-62.
9. Kakuda, R.; Iijima, T.; Yaoita, Y.; Machida, K.; Kikuchi, M., Secoiridoid glycosides from *Gentiana scabra*. *J Nat Prod* **2001,** 64, (12), 1574-5.
10. Zhou, Y.; Di, Y.-T.; Gesang, S.; Peng, S.-L.; Ding, L.-S., Secoiridoid Glycosides from *Swertia mileensis*. *Helvetica Chimica Acta* **2006,** 89, (1), 94-102.
11. Tan, R. X.; Kong, L. D.; Wei, H. X., Secoiridoid glycosides and an antifungal anthranilate derivative from *Gentiana tibetica*. *Phytochemistry* **1998,** 47, (7), 1223-6.

12. Kou, Y.; Yi, X.; Li, Z.; Ai, Y.; Ma, S.; Chen, Q., A Comparative Transcriptomic with UPLC-Q-Exactive MS Reveals Differences in Gene Expression and Components of Iridoid Biosynthesis in Various Parts of *Gentiana macrophylla*. *Genes (Basel)* **2022,** 13, (12).
13. Du, X. G.; Wang, W.; Zhang, Q. Y.; Cheng, J.; Avula, B.; Khan, I. A.; Guo, D. A., Identification of xanthones from Swertia punicea using high-performance liquid chromatography coupled with electrospray ionization tandem mass spectrometry. *Rapid Commun Mass Spectrom* **2012,** 26, (24), 2913-23.
14. Yu, C.; Zhao, Y.; Chen, G., *Swertia pseudochinensis*, a New Plant Source of Andrographolide. *Chemistry of Natural Compounds* **2013,** 49, (1), 119-120.
15. Luo, J.; Yuan, H.; Liang, L.; Xie, Q.; Jiang, S.; Fu, Y.; Chen, S.; Wang, W., An integrated strategy for quality control of the multi-origins herb medicine of Gentianae Macrophyllae Radix based on UPLC-Orbitrap-MS/MS and HPLC-DAD. *RSC Adv* **2023,** 13, (13), 8847-8862.
16. Calis, I.; Ruegger, H.; Chun, Z.; Sticher, O., Secoiridoid Glucosides Isolated from *Gentiana gelida*. *Planta Med* **1990,** 56, (4), 406-9.
17. Ying-Jun, Z.; Chong-Ren, Y., Two triterpenoids from *Gentiana tibetica*. *Phytochemistry* **1994,** 36, (4), 997-999.
18. Zhang, J.; Li, Y.; Li, L.; Zhang, J.-L.; Zhong, L.-W.; Zhang, J.-Y.; Zhong, S.-H.; Gu, R., Study on pharmacodynamic material basis and mechanism of anti-inflammatory effect of Tibetan medicine *Gentiana lawrencei* var. *farreri* based on UPLC-Q-TOF/MS combined with network pharmacology and molecular docking. *Arabian Journal of Chemistry* **2025,** 18, (1).

| | | S-TPC | S-TFC | S-Gentio-picroside | S-Swertia-marin | S-Sweroside | S-FRAP | S-DPPH | R-TPC | R-TFC | R-Gentio-picroside | R-Swertia-marin | R-Sweroside | R-FRAP | R-DPPH |
|---|---|---|---|---|---|---|---|---|---|---|---|---|---|---|---|
| | | Shoots | | | | | | | Roots | | | | | | |
| **Shoots** | **S-TPC [GAE mg/g DW]** | 1.00 | | | | | | | | | | | | | |
| | **S-TFC [QE mg/g DW]** | 0.88 | 1.00 | | | | | | | | | | | | |
| | **S-Gentiopicroside [mg/g DW]** | -0.03 | 0.06 | 1.00 | | | | | | | | | | | |
| | **S-Swertiamarin [mg/g DW]** | -0.75 | -0.83 | -0.32 | 1.00 | | | | | | | | | | |
| | **S-Sweroside [mg/g DW]** | -0.63 | -0.55 | 0.03 | 0.47 | 1.00 | | | | | | | | | |
| | **S-FRAP [TE mg/g DW]** | 0.77 | 0.47 | -0.02 | -0.30 | -0.72 | 1.00 | | | | | | | | |
| | **S-DPPH [TE mg/g DW]** | 0.85 | 0.64 | -0.42 | -0.35 | -0.69 | 0.87 | 1.00 | | | | | | | |
| **Roots** | **R-TPC [GAE mg/g DW]** | 0.98 | 0.89 | -0.14 | -0.74 | -0.50 | 0.67 | 0.83 | 1.00 | | | | | | |
| | **R-TFC [QE mg/g DW]** | 0.71 | 0.85 | -0.07 | -0.71 | -0.21 | 0.16 | 0.44 | 0.79 | 1.00 | | | | | |
| | **R-Gentiopicroside [mg/g DW]** | 0.61 | 0.43 | 0.44 | -0.74 | -0.43 | 0.45 | 0.28 | 0.53 | 0.43 | 1.00 | | | | |
| | **R-Swertiamarin [mg/g DW]** | 0.44 | 0.38 | 0.40 | -0.65 | 0.08 | 0.07 | 0.03 | 0.47 | 0.61 | 0.83 | 1.00 | | | |
| | **R-Sweroside [mg/g DW]** | 0.44 | 0.38 | 0.40 | -0.65 | 0.08 | 0.07 | 0.03 | 0.47 | 0.61 | 0.83 | 1.00 | 1.00 | | |
| | **R-FRAP [TE mg/g DW]** | 0.88 | 0.72 | 0.24 | -0.80 | -0.52 | 0.66 | 0.61 | 0.84 | 0.67 | 0.89 | 0.76 | 0.76 | 1.00 | |
| | **R-DPPH [TE mg/g DW]** | 0.90 | 0.92 | 0.35 | -0.89 | -0.54 | 0.59 | 0.57 | 0.87 | 0.73 | 0.67 | 0.56 | 0.56 | 0.87 | 1.00 |

**Figure S1**. Correlation matrix illustrating relationships between total phenolic content (TPC), total flavonoid content (TFC), antioxidant activity parameters (FRAP, DPPH), and concentrations of selected representative metabolites (gentiopicroside, swertiamarin, sweroside) detected in shoots and roots of somatic hybrids and parental species (*G. cruciata* and *G. tibetica)*. Pearson correlation coefficients statistically significant at $p < 0.05$ are marked with color: green - denoting strong positive correlation, and red - denoting strong negative correlation.
